# Integrative taxonomy of the genus *Onchidium* Buchannan, 1800 (Mollusca, Gastropoda, Pulmonata, Onchidiidae)

**DOI:** 10.3897/zookeys.636.8879

**Published:** 2016-11-24

**Authors:** Benoît Dayrat, Tricia C. Goulding, Deepak Apte, Vishal Bhave, Joseph Comendador, Ngô Xuân Qua,ng, Siong Kiat Tan, Shau Hwai Tan

**Affiliations:** 1 Department of Biology, Pennsylvania State University, University Park, PA 16802, USA; 2 Bombay Natural History Society, Mumbai, Hornbill House, Opp. Lion Gate, Shaheed Bhagat Singh Road, Mumbai 400 001, Maharashtra, India; 3 National Museum of the Philippines, Taft Ave, Ermita, Manila, 1000 Metro Manila, Philippines; 4 Institute of Tropical Biology, Vietnam Academy of Science and Technology, 85 Tran Quoc Toan Str., District 3, Ho Chi Minh city, Vietnam; 5 Lee Kong Chian Natural History Museum, 2 Conservatory Dr, National University of Singapore, 117377, Singapore; 6 Marine Science Laboratory, School of Biological Sciences, Universiti Sains Malaysia, 11800 Minden Penang, Malaysia

**Keywords:** Biodiversity, Euthyneura, Indo-West Pacific, mangroves, marine slugs, South-East Asia

## Abstract

In an effort to clarify the species diversity of onchidiid slugs, the taxonomy of the genus *Onchidium* Buchannan, 1800 is revised using an integrative approach. New, fresh specimens were collected in a large number of places, including type localities. The genus *Onchidium* is redefined here as a clade including only three species which are strongly supported by both morphological and molecular data. All three species were already named: the type species *Onchidium typhae* Buchannan, 1800, *Onchidium stuxbergi* (Westerlund, 1883), and *Onchidium reevesii* (J.E. Gray, 1850). With the exception of a re-description of *Onchidium typhae* published in 1869, all three species are re-described here for the first time. First-hand observations on the color variation of live animals in their natural habitat are provided. The anatomy of each species is described. Important nomenclatural issues are addressed. In particular, *Labella* Starobogatov, 1976 is regarded as a junior synonym of *Onchidium* and *Labella ajuthiae* (Labbé, 1935) and *Onchidium nigrum* (Plate, 1893) are regarded as junior synonyms of *Onchidium stuxbergi*. The nomenclatural status of several other species names is discussed as well. Many new records are provided across South-East Asia and precise ranges of geographic distributions are provided for the genus *Onchidium* and its three species. Distinctive features that help distinguish the genus *Onchidium* from other onchidiids are provided, as well as an identification key for the three species.

## Introduction

The systematics of the Onchidiidae, one of the higher clades of pulmonate gastropods, has been problematic for decades. Many species names were created up to the 1930s, and then the study of their diversity has been more or less abandoned. Identifications have remained nearly impossible at both generic and specific levels. As a result, there are 143 species names and 19 genus names in the literature but the actual species diversity is largely unknown (Dayrat 2009).

The taxonomy of onchidiid slugs has remained problematic primarily because no malacologist has dared to study it in the past 80 years, which in turn is explained by the fact that many serious issues have made onchidiids a nightmare for taxonomists (Dayrat 2009). For instance, most species were described based on preserved specimens with no information on the color and shape of live animals, which turns out to be critical for taxonomic identification; most species were described based on few specimens (and, in fact, a single specimen in many cases), denying individual variation; many type specimens are likely lost; even when types are available, they often were destroyed, with few internal organs left; and, finally, most specimens in museum collections are old and poorly-preserved.

A few years ago, the Dayrat lab embarked on a worldwide systematic revision of the Onchidiidae. Our goal has been to integrate both traditional taxonomy and modern molecular tools (Dayrat 2005). Thanks to local collaborators, thousands of slugs have been collected from 263 stations (as of September 2016) across the tropical Indo-West Pacific. These stations, which include a large number of type localities, are mangrove sites for the most part, although rocky shores and coral rubble areas (where some genera of onchidiids are found) were also visited. Hundreds of individually-numbered slugs were photographed and hundreds of DNA sequences obtained from tissue cuts of those individually-numbered specimens which were also preserved for anatomical dissection. In addition, all the types available were borrowed from museums as well as additional materials, and the most important collections in the world were visited.

Traditionally, especially in museum collections, any onchidiid slug from the tropical Indo-West Pacific was by default referred to as “*Onchidium*,” and any onchidiid slug from outside the tropical Indo-West Pacific was referred to as *Onchidella*. Still to this day, *Onchidella* has not been recorded in the tropical Indo-West Pacific but is found nearly everywhere else except for polar waters (Dayrat et al. 2011b). Also, most authors have agreed that *Onchidella* did not need to be divided into subgroups. Reciprocally, “*Onchidium*” slugs (in the broad sense of non-*Onchidella*) have not been recorded outside the tropical Indo-West Pacific. Because “*Onchidium*” was found to be highly diverse, several generic names were proposed in addition to *Onchidium*, such as *Peronia*, *Paraperonia*, *Paraoncidium*, *Platevindex*, and *Semperoncis*. The application of all these names has remained ambiguous, to say the least (Dayrat 2009).

Our understanding of the onchidiid diversity in the tropical Indo-West Pacific has grown as new data were being gathered. Species diversity, distributions, and higher relationships have become clearer as more and more DNA sequences were obtained and more and more specimens were dissected from new places. As of November 2016, our data set for the onchidiid slugs in the tropical Indo-West Pacific includes approximately 70 species and ten clades of generic level. All species and genera are strongly supported by both DNA sequences and morphology.

After careful examination of all type materials, detailed comparisons between the original descriptions and our own observations, the nomenclatural status of nearly all existing species-group and generic-group names of the Onchidiidae is now known. In other words, it is clear which names are valid names, which names are junior synonyms, which names are *nomina dubia*, and which taxa require new names. So, results on the systematic revision of the Onchidiidae can now be shared.

In a first step, the nomenclature and the alpha-taxonomy of each of the clades of generic level in our data set must be clarified. The present contribution, which focuses on the genus *Onchidium*, marks the beginning of a series of taxonomic papers dedicated to each clade in our data set. Then, in a second step, a phylogenetic tree of the entire family will be provided (still an ongoing endeavor) and that tree used to address broader questions on onchidiid diversification, evolution, and biogeography.

The genus *Onchidium*, the type genus of the family, has been traditionally used by default for many onchidiid species from the tropical Indo-West Pacific. Therefore, it is important to give it a proper definition. The genus *Onchidium* is a clade including only three species, which were already named: the type species, *Onchidium typhae* Buchannan, 1800, with a type locality in Bengal (Ganges delta); *Onchidium stuxbergi* (Westerlund, 1883), with a type locality from Brunei Bay, north-western Borneo; and *Onchidium reevesii* (J.E. Gray, 1850), with a type locality from China (exact locality unknown). With the exception of a re-description of *Onchidium typhae* by Stoliczka (1869), all three species are re-described here for the first time. New synonymies are proposed based on the examination of all available type materials and the careful study of all original descriptions, for the entirety of the Onchidiidae. New geographical records are provided.

Special attention has been given to type localities. Indeed, without going back to type localities to collect fresh specimens, it can be extremely challenging, and often impossible, to address the nomenclatural status of taxon names. For that reason, new specimens have been collected from type localities as far as possible. For the present study, for instance, new specimens of *Onchidium typhae* were collected from the Sundarbans, in West Bengal, India, which corresponds to the type locality, and new specimens of *Onchidium stuxbergi* were collected from Brunei Darussalam, north-western Borneo, extremely close to the type locality.

## Materials and methods

**Collecting.** All specimens examined here were collected by our team, except for the types of existing species and a few specimens found in museum general collections. Local guides (local villagers or fishermen) also often accompanied us. Sites were accessed by car (if next to a road) or by boat (by hiring local fishermen). Local fishermen and villagers are a great source of information to find good collecting sites. They know where to find well-preserved mangroves with old trees and they also know about potential dangers (snakes, crocodiles, wasps, and even tigers in the case of the Sundarbans, West Bengal). Each site was explored for an average of two hours but the exact time spent at each site also depended on the time of the low tide, the weather, etc. At each site, many photographs were taken to keep track of the kind of mangrove being visited (e.g., thick forest of young *Rhizophora* trees, open forest of large *Avicennia* trees) as well as the diverse microhabitats where specimens were collected (e.g., surface of the mud, old and muddy log).

**Specimens.** All available types were examined. Some additional non-type material was collected by others and borrowed from museum collections. However, most specimens were collected by us and our new collections provided fresh material for DNA sequencing and invaluable natural history observations. All our new specimens were deposited in local institutions as vouchers. Acronyms of collections are:

BNHS
Bombay Natural History Society, Mumbai, India 


BDMNH
Brunei Museum, Natural History, Brunei Darussalam 


ITBZC
Institute of Tropical Biology, Zoology Collection, Vietnam Academy of Science and Technology, Ho Chi Minh City, Vietnam 


MNHN
 Muséum national d’histoire naturelle, Paris, France 


NHMUK
Natural History Museum, London, United Kingdom 


PNM
National Museum of the Philippines, Manila, Philippines 


SMF
 Naturmuseum Senckenberg, Frankfurt-am-Main, Germany 


SMNH
Swedish Museum of Natural History, Stockholm, Sweden 


USMMC
Universiti Sains Malaysia, Penang, Malaysia 


ZMB
 Zoologisches Museum, Berlin, Germany 


ZMH
 Zoologisches Museum, Hamburg, Germany 


ZRC
 Zoological Reference Collection, Lee Kong Chian Natural History Museum, National University of Singapore



**Animal preparation and anatomical description.** All anatomical observations were made under a dissecting microscope and drawn with a *camera lucida*. In addition, organs were prepared for scanning electron microscopy (SEM). Radulae were cleaned in 10% NaOH for a week, rinsed in distilled water for at least a week, briefly cleaned in an ultrasonic water bath (less than a minute), sputter-coated with gold-palladium, and examined by SEM. Soft parts (penis and penial hooks) were dehydrated in ethanol and critical point dried before coating. When a lot included several specimens, all pieces of the dissected specimens were carefully numbered, both inside the jar and on the SEM stubs. A range of minimum to maximum animal size is provided for each lot of specimens. In addition, individualized numbers and measurements are provided for the specimens being illustrated here as well as for those comprising our molecular data set. The anatomical description of *Onchidium typhae*, the type species, is fully detailed. The written description of the many anatomical features that are virtually the same between species (nervous system, heart, etc.) is not uselessly repeated three times.

**DNA extraction and PCR amplification.** DNA was extracted using the phenol-chloroform extraction protocol with cetyltrimethyl-ammonium bromide (CTAB). Portions of two mitochondrial genes (COI, 16S) were amplified using the following universal primers: COIF (5’-3’) GGT CAA CAA ATC ATA AAG ATA TTG G, and COIR (5’-3’) TAA ACT TCA GGG TGA CCA AAR AAY CA (Folmer et al. 1994); 16Sar (5’-3’) CGC CTG TTT ATC AAA AAC AT, and 16S 972R (5’-3’) CCG GTC TGA ACT CAG ATC ATG T (Klussmann-Kolb et al. 2008). The 25 µl PCR reactions contained 15.8 µl of water, 2.5 µl of 10X PCR Buffer, 1.5 µl of 25 mM MgCl2, 0.5 µl of each 10 µM primer, 2 µl of dNTP Mixture, 0.2 µl (1 unit) of TaKaRa Taq (Code No. R001A), 1 µl of 20 ng/µl template DNA, and 1 µl of 100X BSA (Bovine Serum Albumin). The thermoprofile used for COI and 16S was: 5 minutes at 94°C; 30 cycles of 40 seconds at 94°C, 1 minute at 46°C, and 1 minute at 72°C; and 10 minutes at 72°C. The PCR products were cleaned with ExoSAP-IT (Affymetrix, Santa Clara, CA, USA) prior to sequencing. Sequenced fragments represented ~680 bp of COI, and ~530 bp of 16S.

**Phylogenetic analyses.** Alignments were obtained using Clustal W in MEGA 6 (Tamura et al. 2013). Chromatograms were consulted to resolve rare ambiguous base calls. DNA sequences were all deposited in Genbank and vouchers clearly identifiable in museum collections (Table 1). The ends of each alignment were trimmed and sequences were concatenated. The concatenated alignment included 993 nucleotide positions: 582 (COI) and 411 (16S). In addition to analyses with the two concatenated markers, another set of analyses was performed with only COI sequences. Pairwise genetic distances between COI sequences were calculated in MEGA 6. Prior to phylogenetic analyses, the best-fitting evolutionary model was selected using the Model Selection option from Topali v2.5 (Milne et al. 2004). A GTR + G + I model was selected. Other (unpublished) analyses were performed using different models, which all yielded identical results. Maximum Likelihood analyses were performed using PhyML (Guindon and Gascuel 2003) as implemented in Topali v2.5. Node support was evaluated using bootstrapping with 100 replicates. Bayesian analyses were performed using MrBayes v3.1.2 (Ronquist and Huelsenbeck 2003) as implemented in Topali v2.5, with four simultaneous runs of 10^6^ generations each, sample frequency of 100, and burn in of 25% (and posterior probabilities were also calculated). Three other onchidiid species and their corresponding COI and 16S sequences were selected from previous studies from our lab as out-groups (Dayrat et al. 2011a: *Onchidella celtica* (Cuvier *in* Audouin and Milne-Edwards 1832), *Peronia* sp. (Okinawa), and *Peronia* sp. (Hawaii)). Other (unpublished) analyses were performed using different combinations of outgroups, which all yielded identical results.

**Table 1.** GenBank accession numbers for COI and 16S DNA sequences. All sequences are new, except for the specimens from China (Sun et al. 2014) and the *Peronia* and *Onchidella* out-groups (Dayrat et al. 2011a). Sun et al. (2014) misidentified all specimens from China as *Onchidium* “*struma*” (*nomen nudum*). Information on individually-identified specimens can be found in the additional material examined (see Fig. 1). The individual numbers starting with “S” from China correspond to vouchers used by Sun et al. (2014).

Species	Individual (DNA)	Locality	GenBank COI	GenBank 16S
*Onchidella celtica*		Ceuta, Northern Africa	AY345048	AY345048
*Onchidella floridana*		Tobago	HQ660035	HQ659903
*Peronia* sp.		Okinawa, Japan	HQ660043	HQ659911
*Peronia* sp.		Hawaii, USA	HQ660038	HQ659906
***Onchidium typhae***	1064	West Bengal, India	---	KX179528
	1089	Andaman, India	KX179512	KX179529
	1109	Andaman, India	KX179513	KX179530
	967	Peninsular Malaysia	KX179510	KX179526
	965	Peninsular Malaysia	KX179509	KX179525
	1007	Singapore	KX179511	KX179527
***Onchidium stuxbergi***	971	Peninsular Malaysia	KX179514	KX179531
	1048	Brunei	KX179515	KX179532
	3251	Bohol, Philippines	KX179517	KX179534
	3363	Bohol, Philippines	KX179518	KX179535
	5602	Vietnam	KX179519	KX179536
	5605	Vietnam	KX179520	KX179537
	S891	China (19°56'N)	JN543155	JN543091
***Onchidium reevesii***	S871	China (22°30'N)	JN543161	JN543097
	S831	China (24°24'N)	JN543160	JN543096
	S853	China (27°29'N)	JN543164	JN543100
	S821	China (33°20'N)	JN543162	JN543098
	S802	China (34°46'N)	JN543157	JN543093

### Phylogenetic results

**Figure 1.**
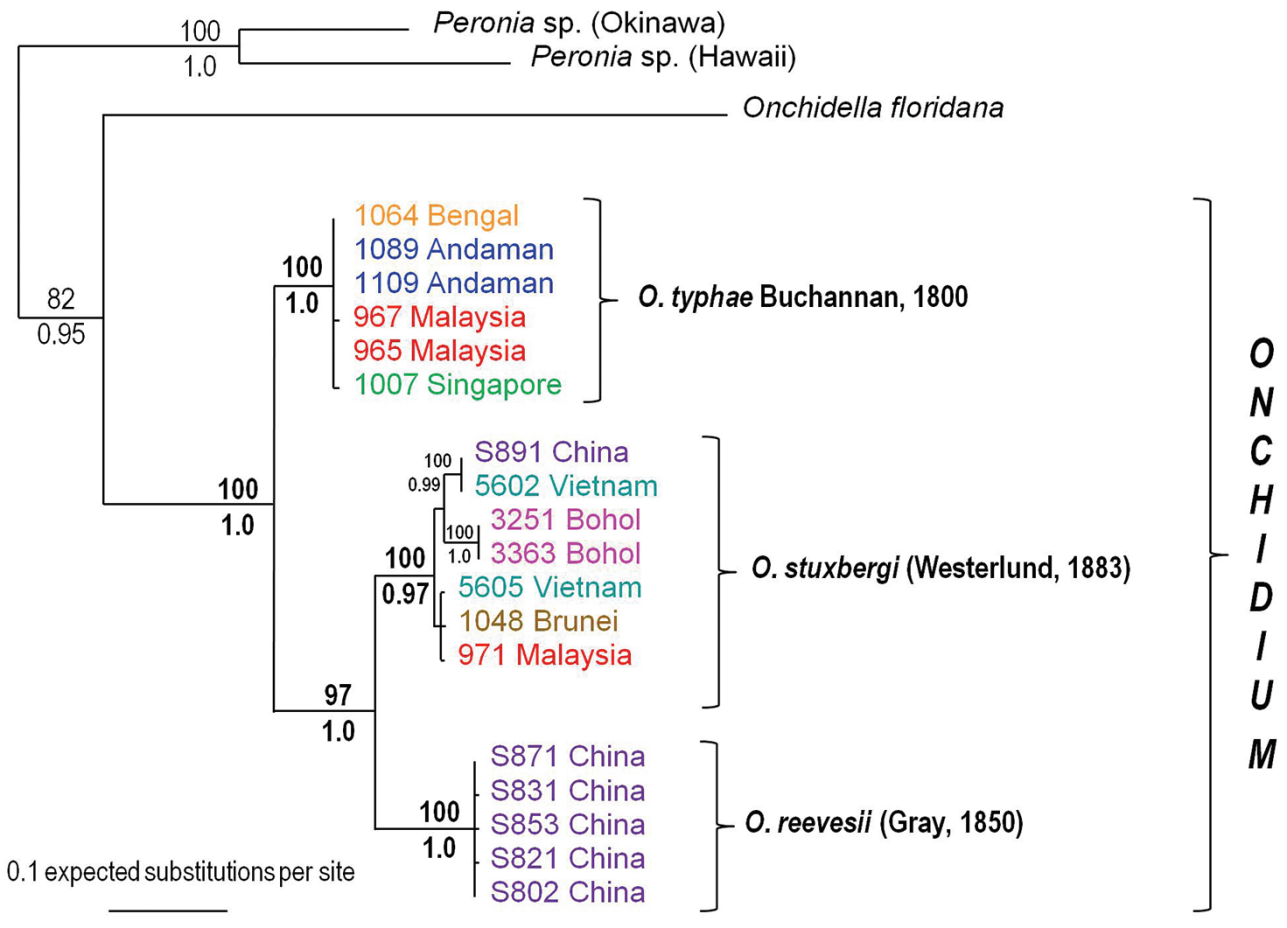
Phylogenetic tree. Relationships within the genus *Onchidium* based on COI and 16S sequences. Numbers above branches are the bootstrap values (Maximum Likelihood analysis) and below are the posterior probabilities (Bayesian analysis); only significant numbers (> 80% and > 0.9) are indicated. *Onchidella* and *Peronia* sequences serve as outgroups. Numbers for each individual correspond to unique identifiers in our DNA collection. All sequences of *Onchidium* specimens are new, with the exception of the specimens from China which were all misidentified as *Onchidium* “*struma*” by Sun et al. (2014). Information on individually-identified specimens can be found in the additional material examined and in Table 1.

**Molecular phylogenetic analyses.** Here, the primary purpose of using DNA sequences is to test the species limits within *Onchidium*. The phylogenetic analyses yielded three species units that are all reciprocally monophyletic and strongly supported (Fig. 1). Each species is supported by a bootstrap support of 100 and posterior probabilities of 1 (except for *Onchidium stuxbergi* with a posterior probability of 0.97). Within *Onchidium typhae* and *Onchidium reevesii*, there is virtually no phylogenetic structure. Within *Onchidium stuxbergi*, the specimens from Bohol (Philippines), cluster together in a well-supported (100/1.0) subunit. This, however, does not warrant any species status to the Bohol specimens because they are nested within *Onchidium stuxbergi* which, without them, would not be monophyletic.

**Pairwise genetic divergences.** The pairwise genetic distances unambiguously support the existence of three species of *Onchidium* (Table 2). There is a wide gap between intra- and inter-specific distances. All intra-specific genetic distances are below 5.1% (below 5.1% within *Onchidium stuxbergi* and below 0.7% for the two other species). All inter-specific genetic distances are minimally 15% (between *Onchidium stuxbergi* and *Onchidium reevesii*) and as high as 28.3% (between *Onchidium typhae* and *Onchidium reevesii*).

**Table 2.** Intra- and inter-species pairwise genetic distances. Ranges of minimum to maximum distances are indicated (in percentage). For instance, within *Onchidium typhae*, individual sequences are between 0% to 0.5% divergent, and individual sequences between *Onchidium stuxbergi* and *Onchidium typhae* are minimally 21.7% and maximally 26.2% divergent.

Species	*Onchidium typhae*	*Onchidium stuxbergi*	*Onchidium reevesii*
***Onchidium typhae***	0.0–0.5		
***Onchidium stuxbergi***	21.7–26.2	0.0–5.1	
***Onchidium reevesii***	26.9–28.3	15.0–18.1	0.0–0.7

## Systematics and anatomical descriptions

### Family Onchidiidae Rafinesque, 1815

#### Genus Onchidium

Taxon classificationAnimaliaSystellommatophoraOnchidiidae

Buchannan, 1800

##### Type species.

*Onchidium typhae* Buchannan, 1800, by monotypy.

*Labella* Starobogatov, 1976: 211. **New synonym.** Type species, by monotypy, *Labella ajuthiae* (Labbé, 1935); replacement name of *Elophilus* Labbé, 1935, preoccupied by *Elophilus* Meigen, 1803 [Diptera].

##### Remarks.

The synonymy of *Labella ajuthiae* (Labbé, 1935) with *Onchidium stuxbergi* (Westerlund, 1883) is discussed in the remarks on *Onchidium stuxbergi*. *Labella* is a junior synonym of *Onchidium* because the two generic names *Labella* and *Onchidium* apply to the same clade. Baker (1938) provided a list of misspellings and unjustified emendations of *Onchidium*: *Onchidion*, *Onchydium*, *Orchidium*, and *Oncidium*.

##### Diagnosis.

Body not flattened. No marginal glands in the notum. No dorsal gills. Dorsal eyes present on notum. Fully retractable, central papilla (with three dorsal eyes) present. Long eye tentacles. Male opening inferior to the right ocular tentacle, slightly to its left. Pneumostome medial. Intestine of types II and III. Rectal gland present. Accessory penial gland and hollow spine present. Penis with hooks.

##### Distinctive diagnostic features.

*Onchidium* differs from all other onchidiids by the presence of unmistakably, large, conical, pointed papillae on the dorsum of live animals. Disturbed live animals and preserved animals are retracted and their dorsal papillae are significantly smaller. The identification to the genus level can then be more challenging.

##### Distribution.

**Figure 2.**
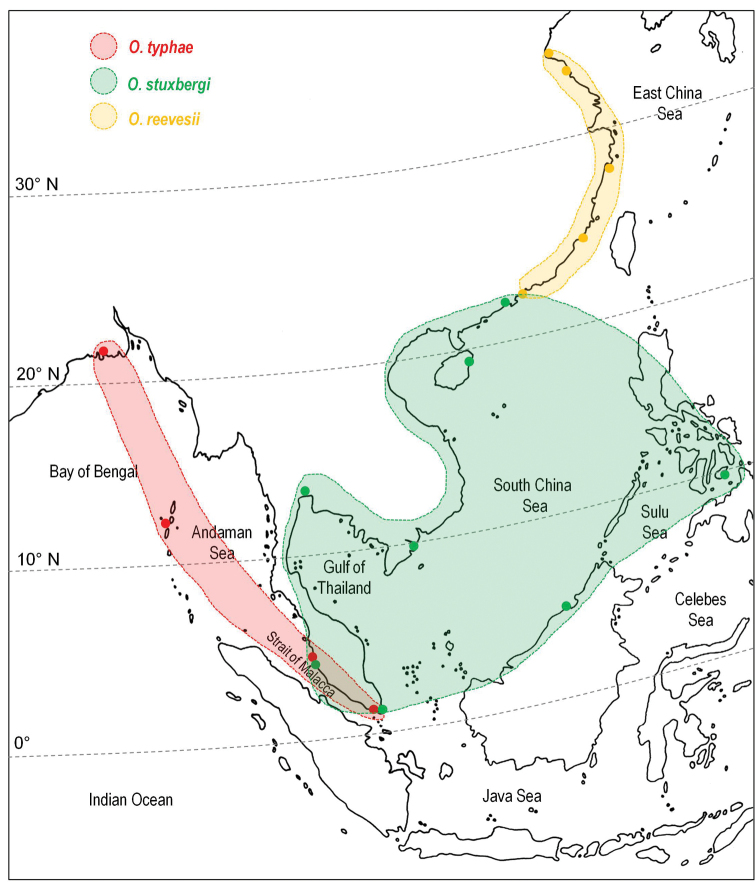
Geographic distribution of the genus *Onchidium* and its three valid species. The colored dots correspond to the known records for each species. Colored areas correspond to hypothetical ranges proposed based on those known records. Naturally, details about the distribution of each species remain uncertain. For instance, *Onchidium typhae* may or may not be found on the western coasts of Thailand by the Andaman Sea.

From north-eastern India (West Bengal) to the Philippines, including the Strait of Malacca, Singapore, Thailand, Vietnam, eastern Borneo, and China (Fig. 2).

#### Onchidium typhae

Taxon classificationAnimaliaSystellommatophoraOnchidiidae

Buchannan, 1800

**Figure 3.**
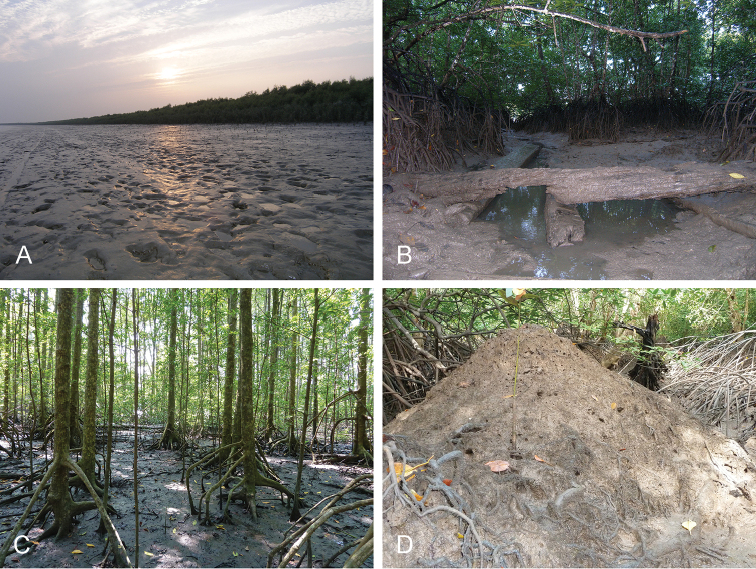
Habitats for *Onchidium typhae*. **A** India, West Bengal, very soft mud on the shore with recently-planted *Avicennia* trees (station 49) **B** India, Andaman Islands, by a large river, deep mangrove with tall trees, small creeks, and plenty of old muddy logs (station 57) **C** Malaysia, Matang, old and open *Rhizophora* forest with tall trees, hard mud, creeks, and many old logs (station 28) **D** Malaysia, Langkawi Island, open forest with high mud lobster (*Thalassina*) mounds (station 26).

**Figure 4.**
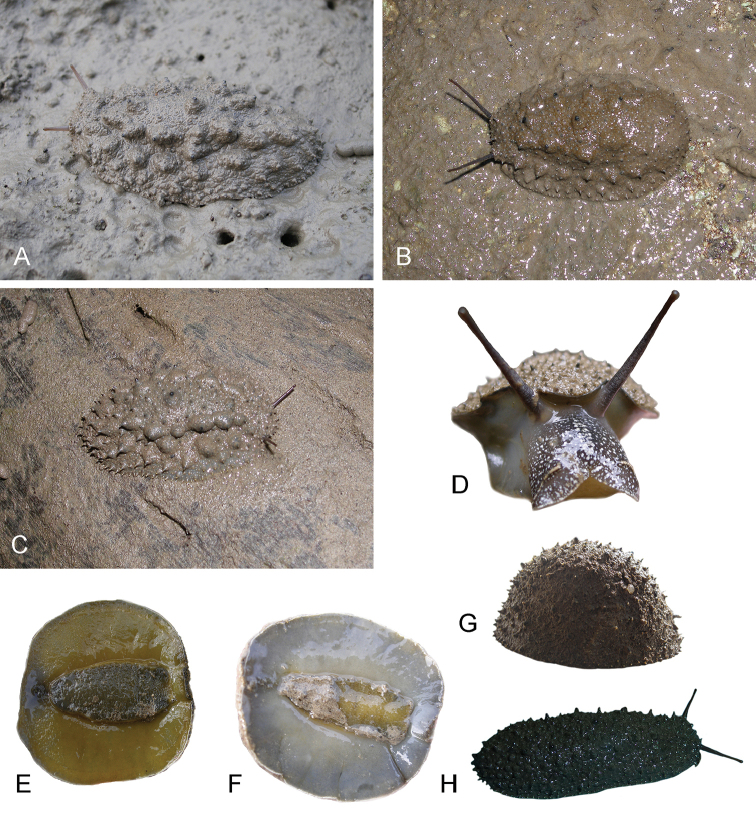
Live specimens, *Onchidium typhae*. **A** Dorsal view, 45 mm long, India, West Bengal, station 48 (BNHS) **B** Dorsal view, 55 mm long, India, Andaman Islands, station 57 (BNHS) **C** Dorsal view, 40 mm long, Singapore (ZRC.MOL.6396) **D** Frontal view, 25 mm wide, India, Andaman Islands, station 57 (BNHS) **E** Ventral view, 50 mm long, India, Andaman Islands, station 59 (BNHS) **F** Ventral view, 40 mm long [DNA 1064], India, West Bengal, station 49 (BNHS) **G** Dorso-lateral view, 40 mm long, India, Andaman Islands, station 59 (BNHS) **H** Dorsal view, 42 mm long, Malaysia, Matang (USMMC 00005, #1).

**Figure 5.**
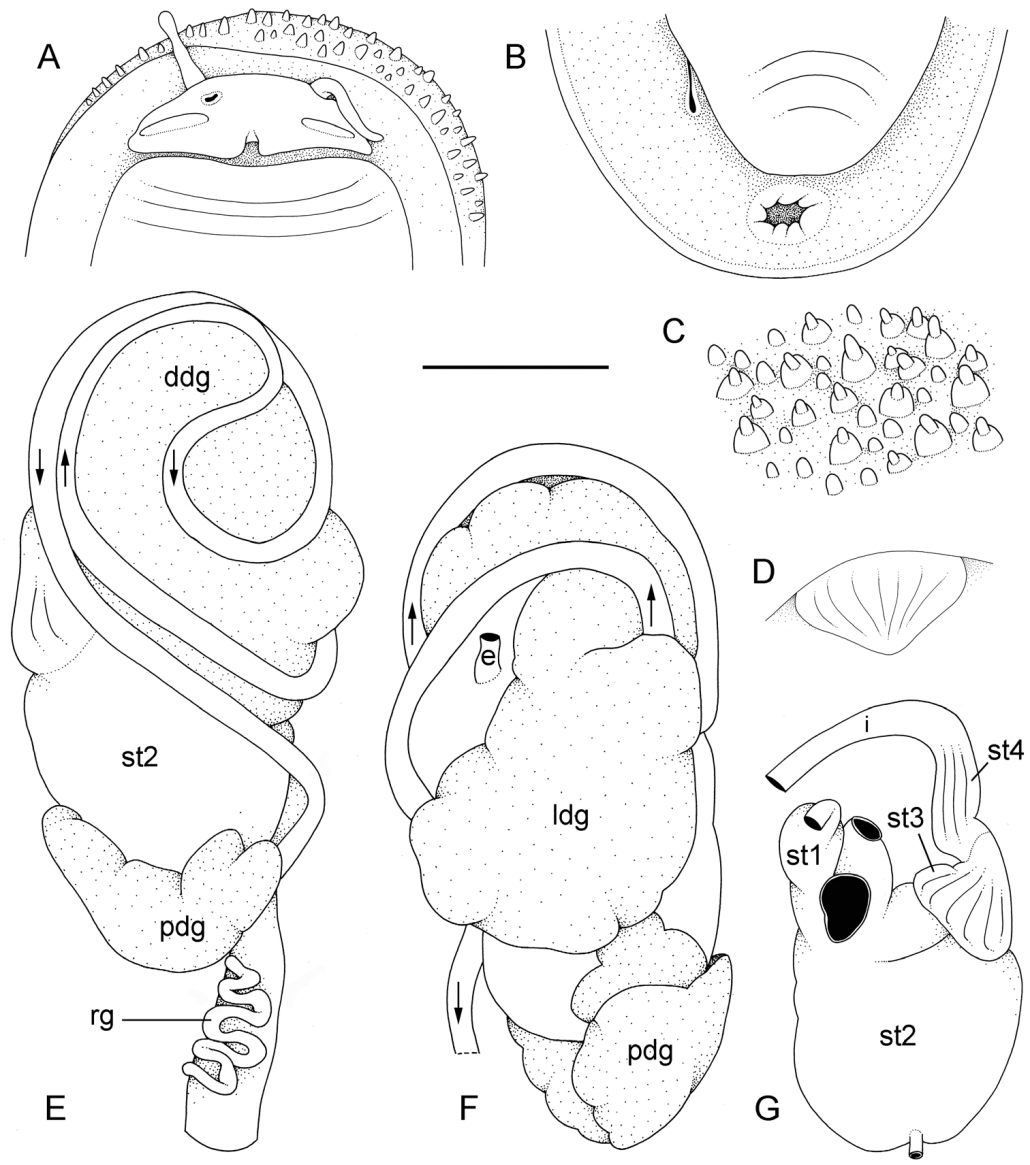
External morphology and digestive system, *Onchidium typhae*. **A** Anterior region, ventral view, Singapore, scale bar 8 mm (ZRC.MOL.6396) **B** Posterior region, ventral view, Singapore, scale bar 7 mm (ZRC.MOL.6396) **C** Dorsal papillae (preserved), Singapore, station 8, scale bar 3.8 mm (ZRC.MOL.6396) **D** Buccal gland, Malaysia, Langkawi Island, scale bar 3 mm (USMMC 00002, #1) **E** Digestive system, dorsal view, Singapore, scale bar 5 mm (ZRC.MOL.6397, #1) **F** Digestive system, ventral view, Singapore, scale bar 5 mm (ZRC.MOL.6397, #1) **G** Stomach (digestive gland removed), ventral view, Singapore, scale bar 5 mm (ZRC.MOL.6397, #1). Abbreviations: **ddg**, dorsal lobe of digestive gland; **e**, esophagus; **i**, intestine; **ldg**, lateral lobe of the digestive gland; **pdg**, posterior lobe of the digestive gland; **rg**, rectal gland; **st1**, stomach chamber 1; **st2**, stomach chamber 2; **st3**, stomach chamber 3; **st4**, stomach chamber 4.

**Figure 6.**
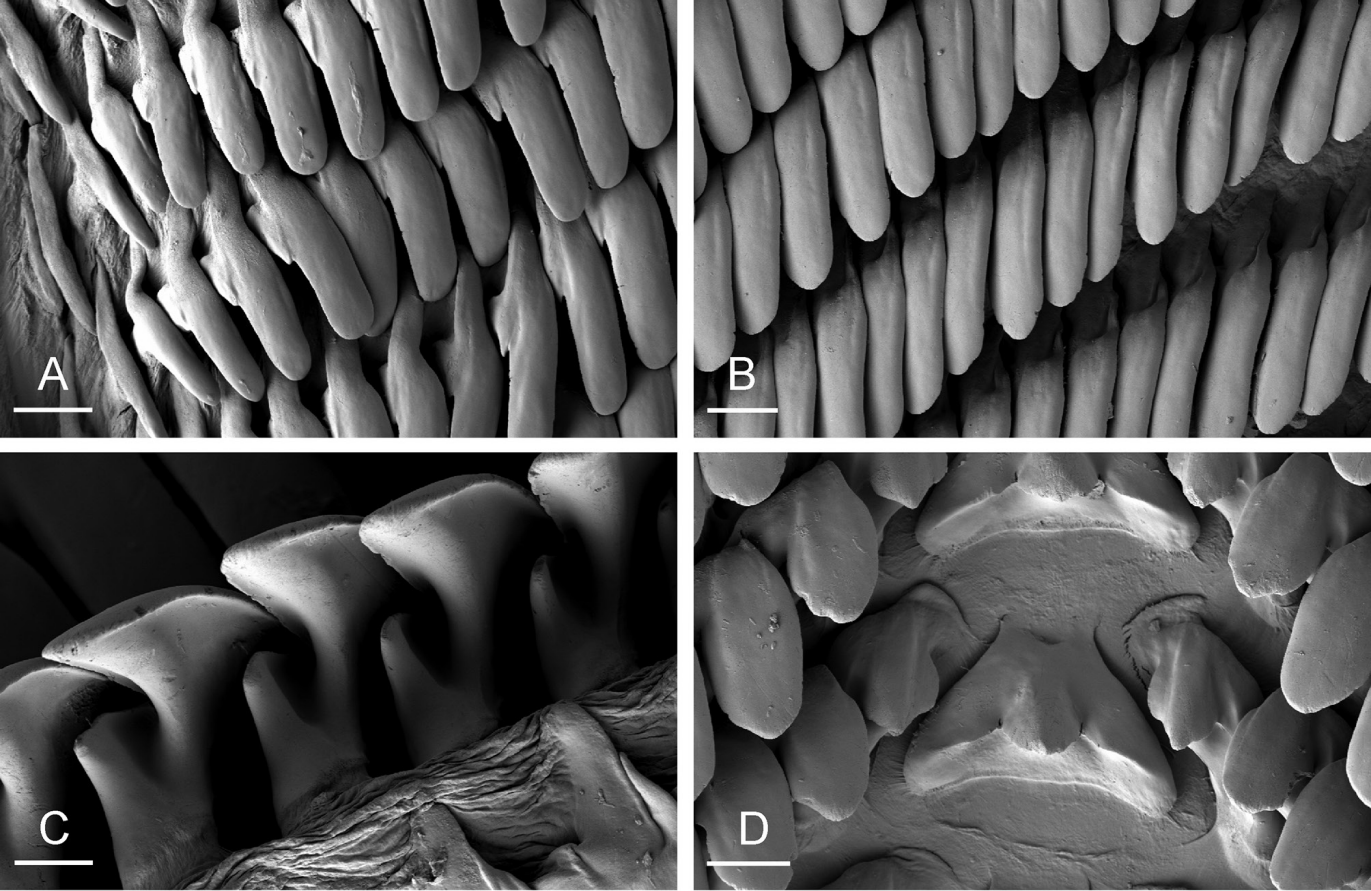
Radula, *Onchidium typhae*, Malaysia, Matang (USMMC 00005, #1). **A** Outermost lateral teeth, scale bar 20 µm **B** Lateral teeth, scale bar 20 µm **C** Lateral teeth, inferior view, showing the additional outer spine of each tooth, scale bar 10 µm **D** Rachidian and innermost lateral teeth, scale bar 20 µm.

**Figure 7.**
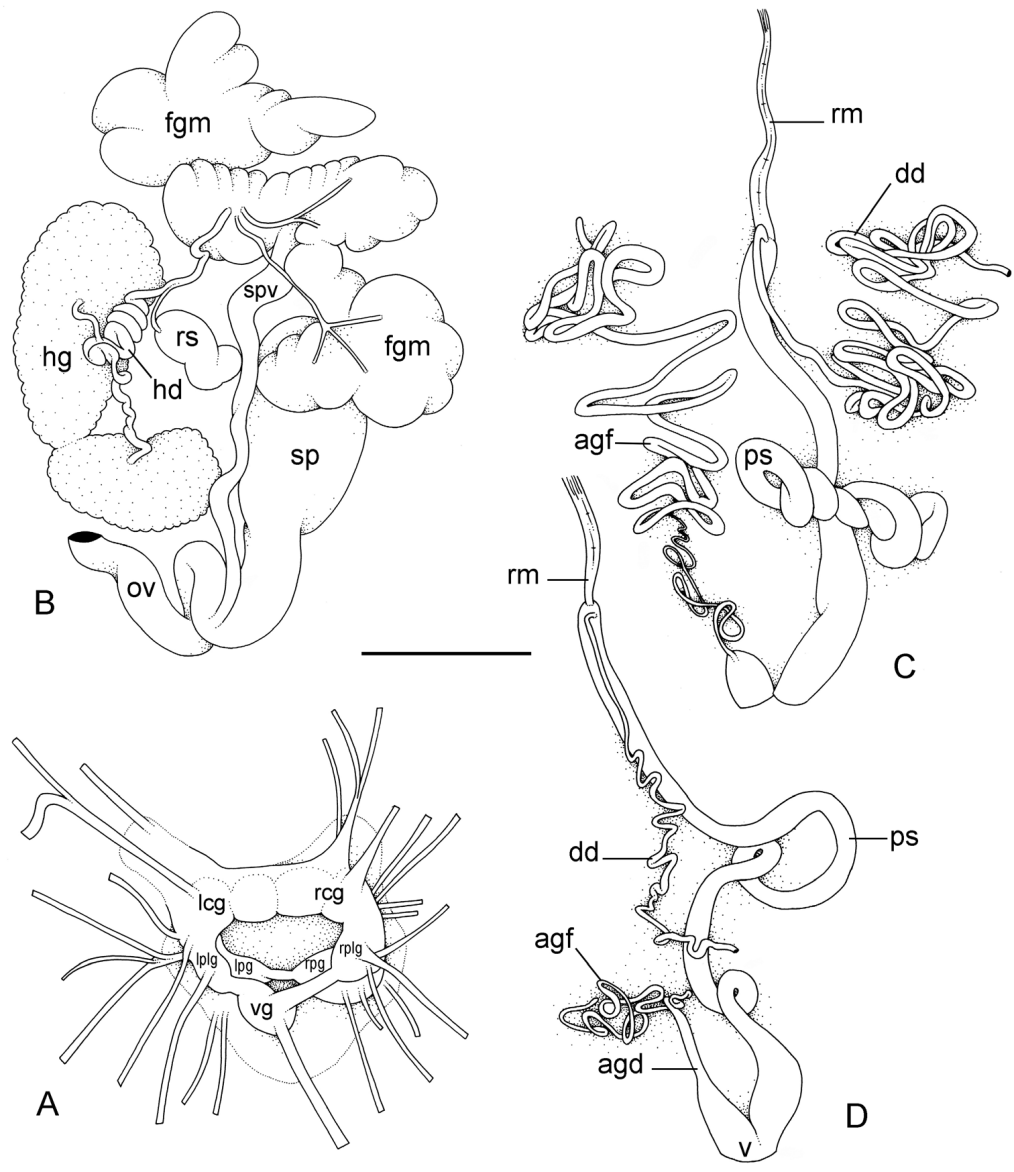
Nervous and reproductive systems, *Onchidium typhae*. **A** Nervous system, Malaysia, Langkawi Island, scale bar 3.8 mm (USMMC 00002, #1) **B** Hermaphroditic (female), posterior parts, Singapore, scale bar 3.8 mm (ZRC.MOL.6397, #1) **C** Male, anterior, copulatory parts, Malaysia, Matang, scale bar 5 mm (USMMC 00005, #1) **D** Male, anterior, copulatory parts, Malaysia, Matang, scale bar 2.7 mm (USMMC 00005, #2). Abbreviations: agf, accessory gland flagellum; dd, deferent duct; fgm, female gland mass; hd, hermaphroditic duct; hg, hermaphroditic gland; lcg, left cerebral ganglion; lpg, left pedal ganglion; lplg, left pleural ganglion; ov, oviduct; ps, penial sheath; rcg, right cerebral ganglion; rm, retractor muscle; rpg, right pedal ganglion; rplg, right pleural ganglion; rs, receptaculum seminis; sp, spermatheca; spv, spermoviduct; v, vestibule; vg, visceral ganglion.

**Figure 8.**
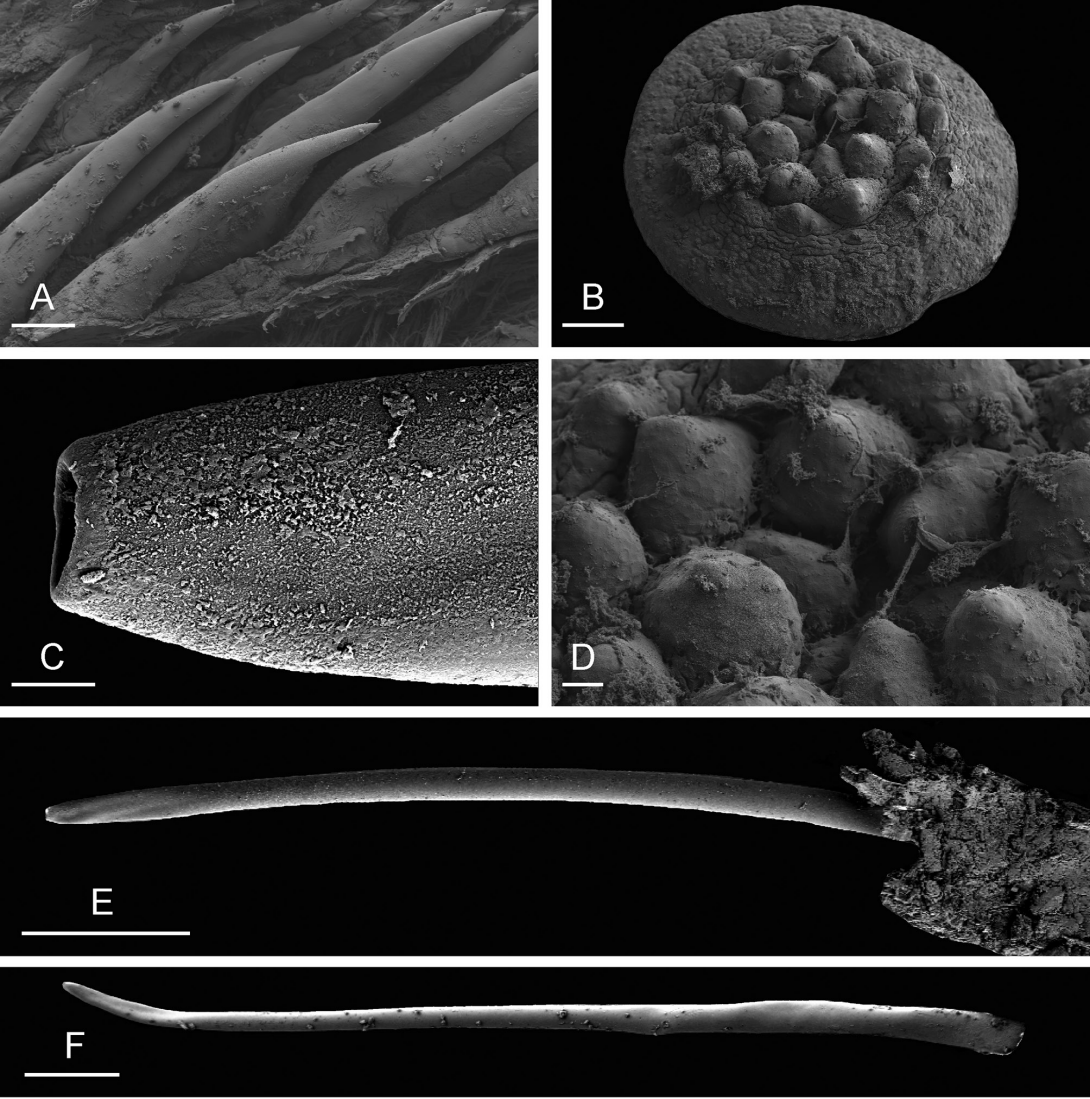
Male, anterior, copulatory parts, *Onchidium typhae*. **A** Penial hooks, Malaysia, Matang, scale bar 30 µm (USMMC 00005, #1) **B** Flat disc at distal end of flagellum of penial accessory gland, Malaysia, Langkawi Island, scale bar 100 µm (USMMC 00002, #1) **C** Tip of hollow spine, penial accessory gland, Singapore, scale bar 4 µm (ZRC.MOL.6397, #1) **D** Detail of B, scale bar 20 µm (USMMC 00002, #1) **E** Hollow spine, penial accessory gland, Singapore, scale bar 100 µm (ZRC.MOL.6397, #1) **F** Hollow spine, penial accessory gland, Malaysia, Langkawi Island, scale bar 100 µm (USMMC 00002, #1).

Figs 3 , 4 , 5 , 6 , 7 , 8

Onchidium typhae Buchannan, 1800: 132–134, plate V, figs 1–3; Stoliczka 1869: 90–103, plate xiv, figs 1–6.

##### Type locality.

“Bengal.” Bengal is a vast region of eastern India (and Bangladesh) around the delta of the Ganges. Collecting specimens in West Bengal was the best that could be done to try to go back to the type locality.

##### Type material.

The original type material could not be located and is likely lost. Given that the identity of *Onchidium typhae* is no longer problematic, there is no need to designate a neotype.

##### Additional material dissected.

**Bangladesh**, Sundarbans, delta, October 1927, 1 specimen [25/15 mm], leg. Konietzko, det. as *Onchidium* (ZMH 27506/2); **India, West Bengal**, Sundarbans, Bally, Datta River, 21°59.277'N, 088°45.213'E, 04 January 2011, 5 specimens (45/25 to 40/25 mm), leg. B. Dayrat & V. Bhave, [station 48, very narrow band of mud with a few sparse *Avicennia* trees, between the edge of the river and the walls protecting the village, no old logs since firewood is a precious resource] (BNHS); Sundarbans, Amlamethi Island, Bidyadhari River, 22°04.923'N, 088°41.882'E, 05 January 2011, 1 specimen (40/30 [DNA 1064] mm), leg. B. Dayrat & V. Bhave, [station 49, very soft mud on the shore with recently-planted *Avicennia* trees; uninhabited island] (BNHS); **India, Andaman Islands**, Middle Andaman, Rangat, Yerrata, Saban, 12°27.451'N, 092°53.792'E, 10 January 2011, 3 specimens (45/20 to 35/20 mm), leg. B. Dayrat & V. Bhave, [station 56, open, impacted mangrove patch by a creek, near village, with medium trees and old logs] (BNHS); Middle Andaman, Rangat, Shyamkund, 12°28.953'N, 092°50.638'E, 11 January 2011, 25 specimens (55/30 to 30/15 mm; 40/20 [DNA 1089] mm), leg. B. Dayrat & V. Bhave, [station 57, by a large river, deep mangrove with tall trees, small creeks, and plenty of old muddy logs, next to a road and a small cemented bridge for creek] (BNHS); Middle Andaman, Shantipur, Kadamtala, 12°19.843'N, 092°46.377'E, 12 January 2011, 25 specimens (65/30 to 30/20 mm; 40/20 [DNA 1109]), leg. B. Dayrat & V. Bhave, [station 58, open area with hard mud and many old logs, next to a mangrove with medium trees] (BNHS); South Andaman, Bamboo Flat, Shoal Bay, 11°47.531'N, 092°42.577'E, 13 January 2011, 7 specimens (50/35 to 40/25 mm), leg. B. Dayrat & V. Bhave, [station 59, open mangrove with medium trees, hard mud, old logs, next to a road and a small cemented bridge for creek] (BNHS); **Malaysia**, Peninsular Malaysia, Merbok, 05°39.035'N, 100°25.782'E, 12 July 2011, 1 specimen (35/24 mm), leg. B. Dayrat & T. Goulding, [station 21, deep *Rhizophora* forest with old, tall trees, hard mud, many small creeks and many old logs] (USMMC 00001); Langkawi Island, Tanjung Rhu, 06°25.771'N, 099°49.436'E, 13 July 2011, 2 specimens (60/35 [#1] and 27/18 mm), leg. B. Dayrat, [station 23, dense forest with young trees, a few creeks] (USMMC 00002); Langkawi Island, Tanjung Rhu, 06°25.317'N, 099°50.106'E, 15 July 2011, 2 specimens (45/40 and 27/17 [DNA 967] mm), leg. B. Dayrat, [station 26, open forest (mostly *Rhizophora*) with high mud lobster mounds] (USMMC 00003); Peninsular Malaysia, Matang, off Kuala Sepatang, Crocodile River, Sungai Babi Manpus, 04°49.097'N, 100°37.370'E, 19 July 2011, 4 specimens (32/20 to 20/17 mm), leg. B. Dayrat & T. Goulding, [station 28, old and open *Rhizophora* forest with tall trees, hard mud, creeks, and many old logs] (USMMC 00004); Matang, close to the jetty, facing fishermen’s village on the other side of river, 04°50.154'N, 100°36.368'E, 20 July 2011, 18 specimens (42/24 [#1] to 28/18 [#2] and 15/10 [DNA 965] mm), leg. B. Dayrat & T. Goulding, [station 29, oldest and open *Rhizophora* forest of tallest and beautiful trees, with hard mud, many creeks, and many old logs] (USMMC 00005); **Singapore**, Semakau Island, 01°12.083'N, 103°45.585'E, 4 April 2010, 1 specimen (40/22 [DNA 1007] mm), leg. B. Dayrat & S. K. Tan, [station 8, artificial, landfill island with low and very dense newly-planted *Rhizophora* trees; muddy areas in between *Rhizophora* patches and coral rubble close to the shore] (ZRC.MOL.6396);, Lim Chu Kang, 01°26.785'N, 103° 42.531'E, 5 April 2010, 3 specimens (40/20, 38/22 [#1], and 19/10 mm), leg. B. Dayrat & S. K. Tan, [station 9, mangrove east of the jetty; open forest with medium trees and medium mud; ended on sun-exposed mudflat outside the mangrove with soft mud; very polluted with trash] (ZRC.MOL.6397).

##### Distribution

(Fig. 2). India: Bengal (type locality; Stoliczka 1869; present study), Andaman Islands (present study). Bangladesh (present study). Singapore (present study). Malaysia (present study).

##### Habitat

(Fig. 3). In West Bengal, *Onchidium typhae* was collected at both sites on soft mud, just next to the river (brackish water) and a few sparse *Avicennia* trees. In the Andaman Islands, it was collected directly on the mud, on the muddy surface (most often at the base) of tree trunks and roots, and on muddy old logs, inside old and deep mangroves with tall trees as well as more open muddy areas; it was also found on the cemented walls of small bridges (under a road next to mangrove). In Singapore, it was collected on muddy old logs. In Malaysia, it was most often found on muddy old logs and on muddy tree trunks and roots, as well as on the surface of the mud (especially that of mud lobster mounds). *Onchidium typhae* is a very cryptic species, especially when it is on the surface of the mud, and one needs to patiently look for it to find it, especially because it is rarely abundant (see below).

##### Abundance.

In the Sundarbans, *Onchidium typhae* was found in two of the four mangrove sites that were visited, but for a total of only six specimens. In the Andaman Islands, it  was found in four of the five mangrove sites visited. The mangrove site where *Onchidium typhae* was not found was not at all muddy but rather, comprised a sandy patch of *Avicennia* by a coral rubble sandy beach. It was especially abundant at two sites (many specimens were observed but not collected), mostly on or near old logs. In Malaysia, *Onchidium typhae* was found in only five of the 18 mangroves that were visited and it was abundant (17 specimens collected) only at one site, which happens to be one of the best, oldest, most pristine, and most diverse mangrove forests we have ever seen anywhere. In Singapore, *Onchidium typhae* was found in three of the five mangroves that were visited (the two sites mentioned above, and one site by the Mandai River where one specimen was collected but ultimately escaped). However, it is rare there (only five specimens collected in total).

##### Color and morphology of live animals

(Fig. 4). Live animals are abundantly covered with mud and the color of their dorsum can hardly be seen at all. In fact, if it were not for their fecal pellets and their long ocular tentacles, they would be really difficult to find because they can be very cryptic. Once the mud is washed out, their dorsum is brownish, with no particular pattern. The color of the foot and of the hyponotum is important because it differs from the other species described here. The foot and the hyponotum vary from gray to yellow in color, and show sometimes even a greenish hue. The color of the foot and the hyponotum may or may not have the same color. It is remarkable that the color of the foot and of the hyponotum of a particular individual can change rapidly, especially when disturbed. The lateral sides of the foot remain dark grey. Distally, the long ocular tentacles are reddish brown. Proximally, they are darker brown and less reddish, speckled with many tiny white dots. The ocular tentacles are extremely long (easily 2 cm when the animal is undisturbed). The ocular tentacles of all *Onchidium* species described here are significantly longer than in other onchidiids, which is a useful character in the field to identify a slug as an *Onchidium*. The head is reddish brown to black, with many white dots evenly distributed.

When animals are not disturbed, the dorsum is typically covered by large pointed papillae that rest on larger, hemispherical bases. Those pointed papillae are unique to *Onchidium* and extremely useful for identification in the field. For a long time (before we realized those slugs were *Onchidium* in the strict sense), we called them the “spiky” slugs to refer to the unique pointed papillae. Those papillae may seem to be arranged in two to four longitudinal ridges (each with five to ten papillae), but this is not the rule. They bear from one to four black “dorsal eyes” at their tip but some papillae do not bear dorsal eyes (especially on the dorsum margin). As in many other onchidiids, there is a central peduncle entirely retractable within the notum. The central peduncle bears three or four “dorsal eyes” but its size is similar to the large pointed papillae (i.e., it is not significantly larger than the other large papillae). The large pointed papillae are surrounded by small papillae as well, which may be rounded or pointed. As soon as the animal is disturbed (by walking on the mud on which it crawls or by touching its dorsum), all dorsal papillae rapidly retract and the animal looks completely different. It then is evenly covered with minute pointed papillae. The body of disturbed animals also is more humped and their ocular tentacles are entirely retracted. One has to patiently wait for a long time for a disturbed slug to relax again (easily 10 or 15 minutes) so the rule is “take a picture first and then touch it!” Interestingly, the appearance of the dorsum of live, disturbed animals is very close to the appearance of preserved animals, which was quite useful when examining type materials of existing species and recognizing them as *Onchidium* species. Crawling individuals can easily measure 30 to 40 mm in length (largest individuals measured 65 mm).

##### External morphology

(Figs 5A–C). Preserved specimens no longer display the distinct color traits seen in live animals. The color of preserved animals is meaningless and uninformative. The body is not flattened. The notum is elongated, occasionally oval. Dorsal gills are absent. The notum is evenly covered by papillae. Large papillae with hemispherical bases are typical for live animals but, in preserved animals, those papillae are pointier and smaller. These larger papillae are surrounded by even smaller papillae. As in live animals, papillae with so-called ‘dorsal eyes’ are present. There is a central, retractable peduncle in the center of the notum, but it can only be seen in live animals in the field. The hyponotum is horizontal. The width of the hyponotum relative to the width of the pedal sole varies among individuals. The width of the hyponotum ranges from about 1/3 to 1/2 its total width, occasionally extending to 4/5 its total width. The anus is posterior, medial, close to the edge of the pedal sole. On the right side (to the left in ventral view), a peripodial groove is present at the junction between the pedal sole and the hyponotum, running longitudinally from the buccal area to the posterior end, a few millimeters from the anus and the pneumostome. The pneumostome is medial. Its position on the hyponotum relative to the notum margin and the edge of the pedal sole varies among individuals but averages in the middle. The position of the female pore (at the posterior end of the peripodial groove) does not vary much among individuals. In the anterior region, the left and right ocular tentacles are superior to the mouth. They are outside if specimens were relaxed before preservation. Otherwise they are retracted. Eyes are located at the tips of the ocular tentacles. Inferior to the ocular tentacles, superior to the mouth, the head bears a pair of oral lobes. On each oral lobe, there is an elongated bump, likely with sensitive receptors. The male aperture (opening of the copulatory complex) is inferior to the right ocular tentacle, slightly to its left (internal) side.

##### Visceral cavity and pallial complex

(Fig. 5D). Marginal glands (found in *Onchidella*) are absent. The anterior pedal gland is oval and flattened, lying free on the floor of the visceral cavity below the buccal mass. The visceral cavity is not pigmented internally and not divided (the heart is not separated from the visceral organs by a thick, muscular membrane). The heart, enclosed in the pericardium, is on the right side of the visceral cavity, slightly posterior to the middle. The ventricle, anterior, gives an anterior vessel supporting several anterior organs such as the buccal mass, the nervous system, and the copulatory complex. The auricle is posterior. The kidney is more or less symmetrical, the right and left parts being equally developed. The kidney is intricately attached to the respiratory complex. The lung is in two left and right, equally-developed, more or less symmetrical parts.

##### Digestive system

(Figs 5E–G, 6). There are no jaws. The left and right salivary glands, heavily branched, join the buccal mass dorsally, on either side of the esophagus. The radula is in between two large postero-lateral muscular masses. Each radular row contains a rachidian tooth and two half rows of lateral teeth of similar size and shape. Examples of radular formulae are: 58 × 73-1-73 in USMMC 00005 #1 (42 mm long), 58 × 65-1-65 in USMMC 00005 #2 (28 mm long), 53 × 80-1-80 in USMMC 00002 #1 (60 mm long), and 65 × 70-1-70 in ZRC.MOL.6397 #1 (38 mm long). The rachidian teeth are tricuspid: the medial cusp is always present; the two lateral cusps, on the lateral sides of the base of the rachidian tooth, are small and inconspicuous. Rachidian teeth tend to be about half the size of the lateral teeth (with a length of the rachidian tooth usually not exceeding 40 µm). The lateral aspect of the base of the rachidian teeth is straight (not concave). The half rows of lateral teeth form an angle of 45° with the rachidian axis. With the exception of the few innermost and few outermost lateral teeth, the size and shape of the lateral teeth do not vary along the half row, nor do they vary among half rows. The lateral teeth seem to be unicuspid with a flattened and curved hook with a rounded tip (the length of the hook is between 50 and 60 µm), but there also is an outer pointed spine on the lateral expansion of the base. In most cases, the basal lateral spine cannot be observed because it is hidden below the hook of the next, outer lateral tooth. It can only be observed when the teeth are not too close (such as in the innermost and outermost regions) or when teeth are placed in an unusual position. The inner and outer lateral aspects of the hook of the lateral teeth are straight (i.e., not wavy and not forming any protuberance).

The esophagus is narrow and straight, with thin internal folds. The esophagus enters the stomach anteriorly. Only a portion of the posterior aspect of the stomach can be seen in dorsal view because it is partly covered by the lobes of the digestive gland. The dorsal lobe is mainly on the right. The left, lateral lobe is mainly ventral. The posterior lobe covers the posterior aspect of the stomach. The stomach is a U-shaped sac divided in four chambers. The first chamber, which receives the esophagus, is delimited by a very thin layer of tissue, and receives the ducts of the dorsal and lateral lobes of the digestive gland. The second chamber, posterior, is delimited by a thick muscular tissue and receives the duct of the posterior lobe of the digestive gland. It appears divided externally but consists of only one internal chamber. The third, funnel-shaped chamber is delimited by a thin layer of tissue with high ridges internally. The fourth chamber is continuous and externally similar to the third, but it bears only low, thin ridges internally. The intestine is long, narrow, and of type II. A rectal gland is present. It is a long, narrow, and coiled tube that opens in the left portion of the pulmonary complex. Its function is unknown.

##### Nervous system

(Fig. 7A). The circum-esophageal nerve ring is post-pharyngeal and pre-esophageal. The two cerebral ganglia touch and the cerebral commissure is short. However, the length of the cerebral commissure does vary among individuals. Pleural and pedal ganglia are also all distinct. The visceral commissure is short but distinctly present and the visceral ganglion is more or less medial. Cerebro-pleural and pleuro-pedal connectives are short and pleural and cerebral ganglia touch each other. Nerves from the cerebral ganglia innervate the buccal area and the ocular tentacles, and, on the right side, the penial complex. Nerves from the pedal ganglia innervate the foot. Nerves from the pleural ganglia innervate the lateral and dorsal regions of the mantle. Nerves from the visceral ganglia innervate the visceral organs.

##### Reproductive system

(Fig. 7B). Sexual maturity is correlated with animal length. Mature individuals have large female organs (with a large female gland mass) and fully-developed, male, copulatory parts. Immature individuals may have inconspicuous female organs (or simply no female organs at all) and rudimentary anterior male parts. The hermaphroditic gland is a single mass, joining the spermoviduct through the hermaphroditic duct (which conveys the eggs and the autosperm). There is a large, bent, and approximately oval receptaculum seminalis (caecum) along the hermaphroditic duct. The female gland mass contains various glands (mucus and albumen) which can hardly be separated by dissection and of which the exact connections remain uncertain. The hermaphroditic duct becomes the spermoviduct (which conveys eggs, exosperm, and autosperm) which is not divided proximally, at least not externally. A prostate, not distinct externally, may be located within the walls of the spermoviduct. The spermoviduct is completely embedded within the female gland mass, at least proximally. Distally, the spermoviduct branches into the deferent duct (conveys the autosperm up to the anterior region, running through the body wall) and the oviduct. The free oviduct conveys the eggs up to the female opening and the exosperm from the female opening up to the fertilization chamber, which should be near the proximal end of the spermoviduct. The ovate-spherical spermatheca (for the storage of exosperm) connects to the oviduct through a wide and strong duct. The oviduct is narrow, only slightly elongated and convoluted. The vaginal gland is absent.

##### Copulatory apparatus

(Figs 7C–D, 8). The male anterior organs consist of the penial complex (penis, penial sheath, vestibule, deferent duct, retractor muscle) and penial accessory gland (flagellum, and hollow spine). The penial complex and the penial accessory gland share the same vestibule and the same anterior male opening.

The penial gland is a long, tube-like flagellum with a proximal dead end. The length of the flagellum of the penial gland varies among individuals but it is always heavily coiled. Distally, the flagellum ends in a hard, hollow spine protected by a sheath which is fused distally with the vestibule. The hollow spine is narrow and elongated, slightly curved. Its diameter is between 20 and 30 µm but it narrows down distally. The diameter of the opening at its tip is between 4 and 6 µm. Its length ranges from 0.7 mm (ZRC.MOL.6397 #1) to 1.2 mm (USMMC 00005 #1). The hollow spine does not open directly into the vestibule. Instead, the end of the tube of the accessory gland is a disc which is more or less flat (between 0.4 and 0.6 mm in diameter) and bears approximately 20 conical papillae in its center. The hollow spine thus must go through that disc in order to be outside and shared with the partner.

The penial sheath is long and very strongly coiled in spirals. In less mature individuals, the coils may not be as strong and numerous but they are present. The penial sheath protects the penis for its entire length. The insertion of the retractor muscle marks the separation between the penial sheath (and the penis inside) and the deferent duct. The retractor muscle is shorter than the penial sheath and runs straight to the posterior half of the visceral cavity. The insertion of the retractor muscle varies among individuals: in the posterior half of the visceral cavity in all specimens but those from India; in the anterior half of the visceral cavity (just anterior to the heart) in the specimens from India. The deferent duct also is highly convoluted with many loops. In immature specimens, the deferent duct is significantly less coiled. The penis is elongated, round, narrow, and hollow; its diameter is less than 200 µm, and its distal part covered with hooks. When the penis is retracted inside the penial sheath, the hooks are inside the tube-like penis. During copulation, the penis is exerted like a glove and the hooks are then on the outside. Hooks are very densely packed inside the penis, with multiple, irregular rows of an average of 15 hooks around the circumference of the penis. Hooks are conical, slender, sharply pointed, and measure up to 300 µm in length.

##### Distinctive diagnostic features.

Externally, *Onchidium typhae* differs from other *Onchidium* species by the color of the hyponotum, which is not white but instead varies between grayish, yellowish, and even greenish (see below the dichotomous identification key, before the final conclusion). The color of the foot (yellowish, not bright orange) is not diagnostic. Internally, the spirally coiled penial sheath is not diagnostic. However, *Onchidium typhae* is the only *Onchidium* species known so far with an intestine of type II.

##### Remarks.

The original description of *Onchidium typhae* by Buchannan was brief but it was based on first-hand observation of live animals, which is quite unusual for onchidiids since most onchidiid species were described based on preserved material with no information on shape and color of live animals. Even though the type material is likely lost, two features described and illustrated by Buchannan support the identification of the material described here as *Onchidium typhae*. First, and most importantly, the long eye tentacles (only *Onchidium* species have such long eye tentacles) and the dorsal papillae of various sizes (Buchannan’s “glandular tubercles”). Buchannan’s (1800: fig. 2) illustration of an elongated body of a crawling slug is perfectly compatible with what we observed in the field. Buchannan originally described *Onchidium typhae* as non-hermaphroditic; however, as pointed out by Cuvier (1804) shortly after Buchannan’s article, onchidiid slugs are in fact hermaphroditic.

Stoliczka (1869) re-described *Onchidium typhae* based on live animals that he collected himself near Calcutta. His identification is in agreement with Buchannan’s original description. Stoliczka provided some information on the internal anatomy, which is compatible with our observations. Semper (1885) examined two specimens from Calcutta which he identified as *Onchidium typhae*. According to Buchannan (1800), *Onchidium typhae* is found on leaves of *Typha elephantina* (hence the specific name), a common reed in brackish waters of the Ganges delta. Wild areas with reeds have become very rare because Bengal was heavily developed in the last century and most coastal areas were converted to rice fields. According to Stoliczka (1869), however, *Onchidium typhae* is also found “about old bricks” and “in ditches.” Stoliczka also mentioned that it was the only species found near Calcutta, suggesting that in the past it could be found in brackish waters extending far inland. Finally, Stoliczka mentions *Onchidium typhae* from the banks of the Hooghly River, which is very close to where our own specimens were collected in West Bengal.

Semper accepted Stoliczka’s anatomical re-description of *Onchidium typhae* and added some detail on the anterior male parts. In particular, Semper illustrated some penial hooks and the spine of the penial accessory gland. However, the sizes described by Semper (a maximum size of 170 µm for the penial hooks and a length of 4.5 mm for the spine of the penial accessory gland) are not really compatible with the sizes observed for the present study (penial hooks up to 300 µm and a spine less than 1.2 mm long). Therefore, Semper likely examined individuals of a different species, which cannot be identified at this stage.

Hoffmann (1928) re-described a specimen that he identified as *Onchidium typhae*. That identification as an *Onchidium* is possibly correct (because of the presence of a rectal gland and of a penial accessory gland). However, the specimen examined by Hoffmann came from an unknown locality and it remains unclear whether Hoffmann did actually examine *Onchidium typhae*. Labbé (1934) simply mentions the name *Onchidium typhae* with no additional description or records. Dey (2006) illustrated a preserved (and not relaxed) specimen identified as *Onchidium typhae* from the Sundarbans. The identification is possibly correct (the photograph is fuzzy). However, it is unclear whether the brief comments on the natural history of *Onchidium typhae* refer only to *Onchidium typhae* or a mix of species because Dey claims that *Onchidium typhae* is found climbing on the trees, which is uncertain. Based on our observations, *Onchidium typhae* can be found on muddy old logs, but not actually climbing on trees.

Finally, a search for potential synonyms of *Onchidium typhae* revealed no synonyms (the available type materials of all onchidiid species were personally examined) and it does seem that it was named only once.

#### Onchidium stuxbergi

Taxon classificationAnimaliaSystellommatophoraOnchidiidae

(Westerlund, 1883) comb. n.

**Figure 9.**
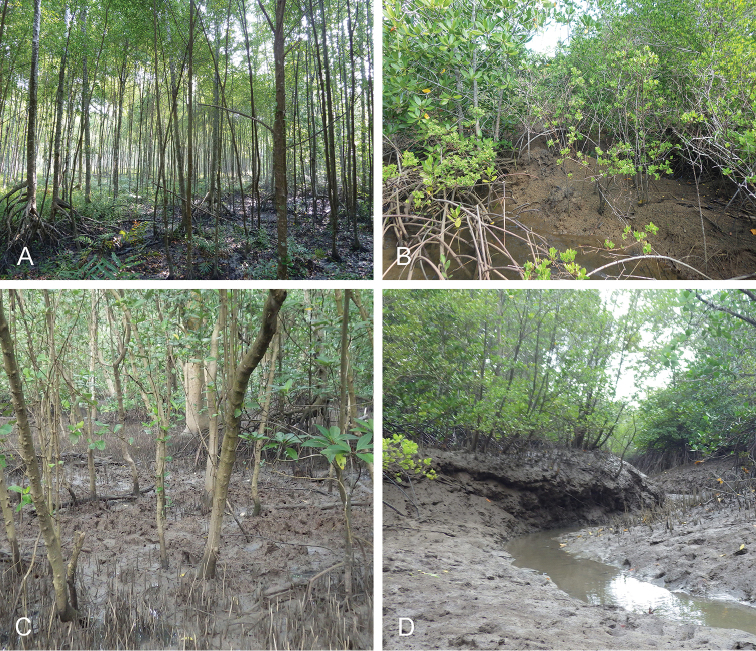
Habitats for *Onchidium stuxbergi*. **A** Malaysia, Kuala Sepatang, old forest with tall, old *Rhizophora* trees, high in the tidal zone (station 27) **B** Philippines, Bohol, mostly *Nypa* palms with *Thalassina* mounds (station 187) **C** Vietnam, Can Gio, open mangrove with large *Avicennia* trees, soft mud, some old logs (station 231) **D** Vietnam, Can Gio, hard mud with trees spread out by a small road and then a steep bank to the soft, deep mud (station 221).

**Figure 10.**
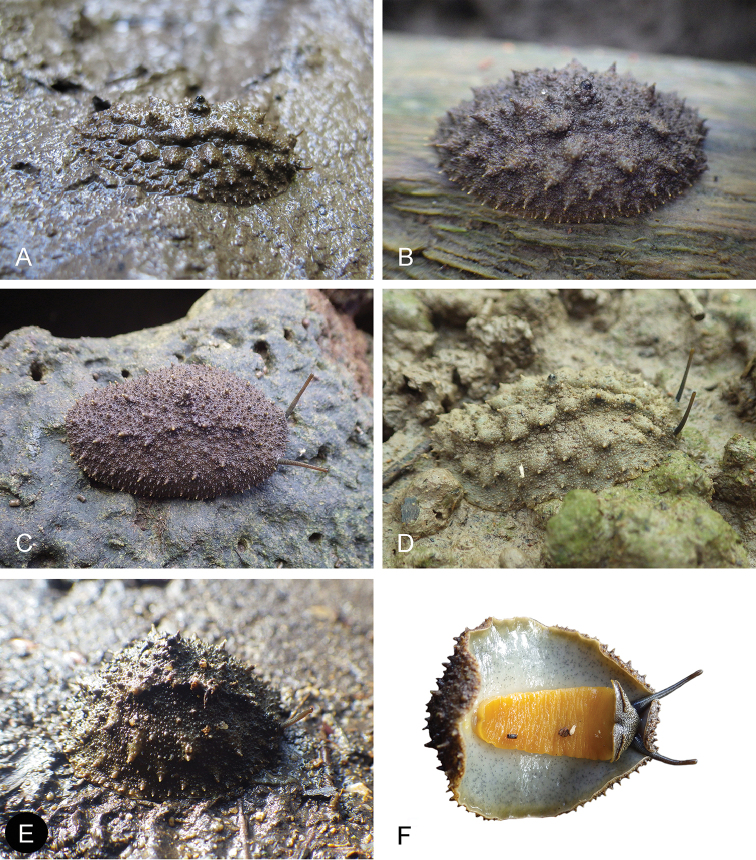
Live specimens, *Onchidium stuxbergi*. **A** Dorsal view, 33 mm long, Brunei Darussalam (BDMNH, #1) **B** Dorsal view, 27 mm long, Philippines, Bohol (PNM 041200) **C** Dorsal view, 35 mm long, Bohol, Philippines (PNM 041201) **D** Dorsal view, 35 mm long, Vietnam, Can Gio (ITBZC IM 00002, #1) **E** Dorsal view, 30 mm long [DNA 3251], Bohol, Philippines (PNM 041199) **F** Ventral view, 35 mm long [DNA 3363], Bohol, Philippines (PNM 041202, #2).

**Figure 11.**
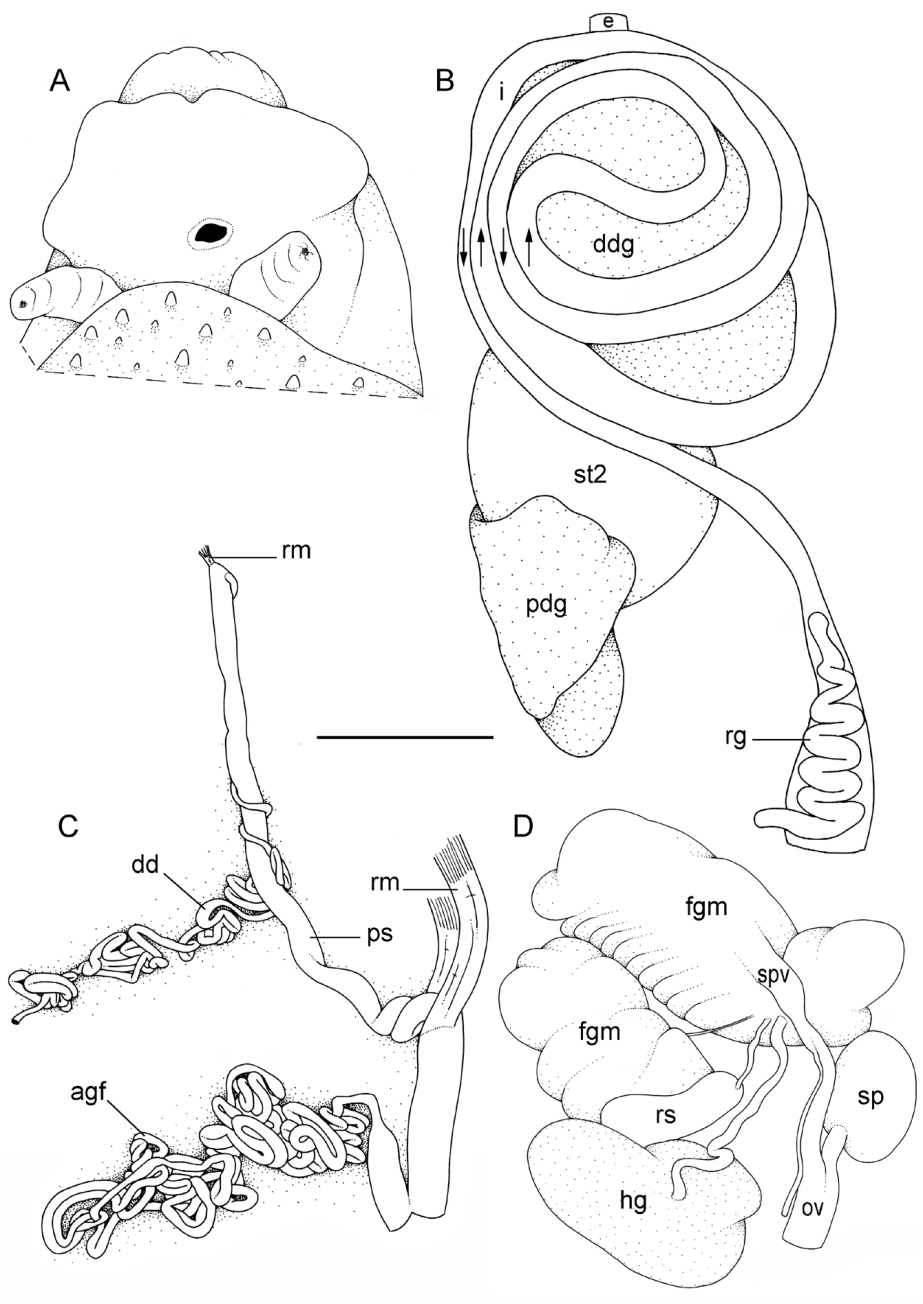
External morphology and internal anatomy, *Onchidium stuxbergi*. **A** Anterior region, dorsal view, Malaysia, Kuala Sepatang, scale bar 4.4 mm [DNA 971] (USMMC 00006) **B** Digestive system, dorsal view, Philippines, Bohol, scale bar 5 mm [DNA 3363] (PNM 041199, #2) **C** Reproductive system, anterior, male, copulatory parts, Philippines, Bohol, scale bar 6.8 mm [DNA 3363] (PNM 041199, #2) **D** Reproductive system, hermaphroditic (female) posterior parts, Malaysia, Kuala Sepatang, scale bar 5 mm [DNA 971] (USMMC 00006). Abbreviations: agf, accessory gland flagellum; dd, deferent duct; ddg, dorsal lobe of digestive gland; e, esophagus; fgm, female gland mass; hg, hermaphroditic gland; i, intestine; ov, oviduct; pdg, posterior lobe of the digestive gland; ps, penial sheath; rg, rectal gland; rm, retractor muscle; rs, receptaculum seminis; sp, spermatheca; spv, spermoviduct; st2, stomach chamber 2.

**Figure 12.**
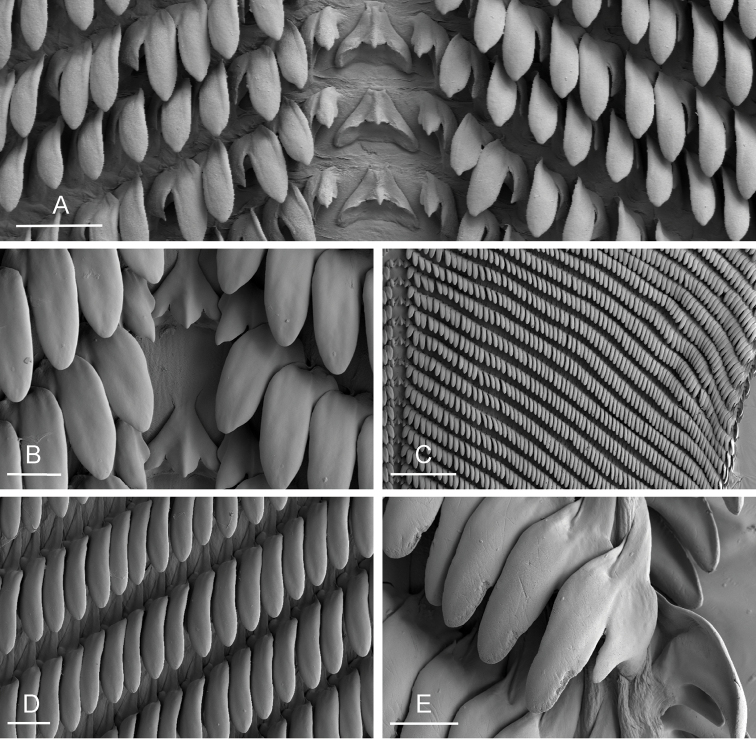
Radula, *Onchidium stuxbergi*. **A** Rachidian and innermost lateral teeth, Brunei Darussalam, scale bar 40 µm (BDMNH # . **B** Rachidian and innermost lateral teeth, Philippines, Bohol, scale bar 20 µm (PNM 041202 #1) **C** Half rows, Philippines, Bohol, scale bar 200 µm (PNM 041202 #1) **D** Lateral teeth, Philippines, Bohol, scale bar 30 µm (PNM 041202 #1) **E** Outermost lateral teeth, Philippines, Bohol, scale bar 20 µm (PNM 041202 #1).

**Figure 13.**
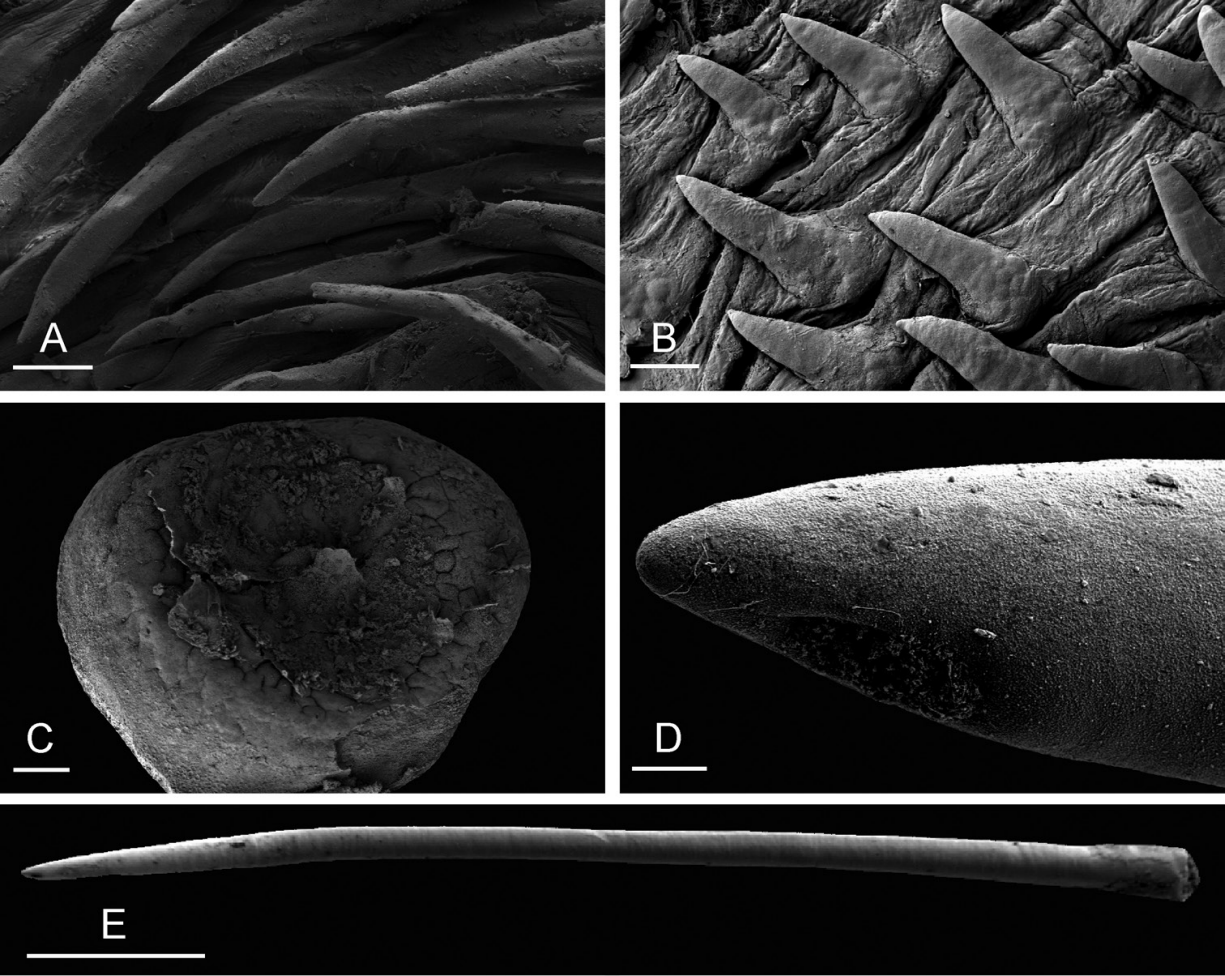
Male, anterior, copulatory parts, *Onchidium stuxbergi*. **A** Penial hooks, Philippines, Bohol, scale bar 40 µm [DNA 3363] (PNM 041199, #2) **B** Penial hooks, Malaysia, Kuala Sepatang, scale bar 20 µm [DNA 971] (USMMC 00006) **C** Flat disc at distal end of flagellum of penial accessory gland, Malaysia, Kuala Sepatang, scale bar 40 µm [DNA 971] (USMMC 00006) **D** Tip, hollow spine, penial accessory gland, Philippines, Bohol, scale bar 3 µm [DNA 3363] (PNM 041199, #2) **E** Hollow spine, penial accessory gland, Philippines, Bohol, scale bar 200 µm [DNA 3363] (PNM 041199, #2).

Figs 9 , 10 , 11 , 12 , 13

Vaginulus stuxbergi Westerlund, 1883: 165; Westerlund 1885, p.191–192, pl. 2, fig. 2a–c.
Oncidium nigrum Plate, 1893: 188–190, pl. 8, fig. 31a, pl. 10, fig. 53, pl. 11, fig. 75; Hoffmann 1928: 78; Labbé 1934: 223–224, figs. 58–61. **New synonym**.
Elophilus ajuthiae Labbé, 1935: 312–317, figs 1–3. **New synonym**.

##### Type locality

**(*Vaginulus stuxbergi*).** “Borneo in silva, ad flum Kalias” means that the slugs were found in forests by a river now called the Klias River. The latter runs into the Brunei Bay, which is a small bay bordered by Brunei Darussalam in the South, by Sabah (Malaysia) in the north, and by the small island of the Labuan Territory (Malaysia) in the west. Several of the labels of the type material indicate Labuan as the locality. So, it is possible that the type material is a mix of specimens collected at Labuan Island itself and on the shore of Borneo, facing Labuan. Here is what the different labels read for the first jar: “*Vaginulus stuxbergi* Westerlund, 1885. Borneo, Labuan. On the beach, mangroves. Leg. Vega Exp 1878-1880, sta. 633. SMNH-Type-7523-syntype(s);” “*Onchidium*. Mangrover Sump, Labuan vid Borneo [i.e., meaning Labuan opposite (seeing) Borneo], Vega Exp. n° 633, 18/11 1879;” and “*Oncis stuxbergi* Wstld, 1883. Hab. Labuan b. Borneo (Mangrove - Sumpf). Leg Vega-Expedition (N°633) 18-xi-1879, Jena, Jan 1927, Hoffmann determ.” Here is what the different labels read for the second jar: “*Vaginulus stuxbergi* Westerlund, 1885. Borneo, Labuan. On the beach, mangroves. Leg. Vega Exp 1878-1880, sta. 633. SMNH-Type-1334-syntype(s);” “*Vaginulus Stuxbergi*;” “*Vaginulus Stuxbergi* Borneo Vega Exp, det. Westerlund;” and “*Oncis stuxbergi* Westerlund, 1883 [Typ fur Vaginula stuxbergi Wsterld]. Hab. Borneo. Leg. Vega-Expedition, Westerlund det. Jena, Jan 1927, Hoffmann determ.” Our specimens here were collected from Brunei Darussalam, which borders the Brunei Bay and faces the island of Labuan, i.e., from a locality that is extremely close to the type locality.

##### Type locality

**(*Onchidium nigrum*).** “Borneo” is the only geographic information provided by Plate (1893) in the original description as well as on the label of the type, which reads “*Oncidium nigrum* Plate. 22749. Borneo. Gera S.” The mention of “Gera S.” does not refer to Sungai Geras, a river near the city of Bintulu, on the west coast of Borneo, in Sarawak, Malaysia, but most likely to the fact that the specimen was collected by Gerard, as indicated in Plate’s (1893: 188) original description (“1 Exemplar von Borneo, durch Gerard gesammelt”). Thus, the type locality of *Onchidium nigrum* could be anywhere in Borneo.

##### Type locality

**(*Elophilus ajuthiae*).** The “Province d’Ajuthia (Siam)” is the province of Ayutthaya, in Thailand, approximately 80 kilometers north of Bangkok, which used to be the capital of the Kingdom of Siam. Because that province is inland, Labbé assumed that the slugs had been collected in fresh water. However, the Chao Phraya River of the basin than runs through the province of Ayutthaya actually is under the influence of the tide year round. The salt front (brackish water) goes up to 75 and 175 kilometers from the river mouth in the wet and dry seasons, respectively, and it was even more so the case in the past when the river side was still not developed (syntypes collected by M. Bocourt in 1862 according to the label of the type material). In the province of Ayutthaya, the river is approximately at its kilometer 140 (Singkran 2015: 28). The label of the type specimens of *Elophilus ajuthiae* says that it lived in the “eaux dormantes de la province d’Ajuthia.” The French expression “eaux dormantes” means “swamps.” Given what is known of the basin of Chao Praya River, those swamps were brackish water under the influence of daily tides.

##### Type material

**(*Vaginulus stuxbergi*).** One lectotype hereby designated (43/25 mm; entire and never dissected; SMNH 1334). All eleven other syntypes become paralectotypes with no name-bearing status. Originally, the type material included a total of twelve specimens split in two different jars: five specimens (all paralectotypes) with catalog number SMNH 7523 (from 35/30 to 15/12 mm, all entire except one specimen opened by a previous investigator, with a vial including the male copulatory system); and seven specimens with catalog number SMNH 1334 (the lectotype 43/25 mm, entire, and six paralectotypes from 20/18 to 15/15 mm, all entire except for one specimen dissected by a previous investigator, with a vial including a male copulatory system). All paralectotypes are *Platevindex* and the lectotype clearly is an *Onchidium*. The only one *Onchidium* specimen was selected as the lectotype because it is the only specimen that Westerlund (1885) illustrated for his new species *Vaginulus stuxbergi*. In fact, Westerlund’s figures are a perfect image of the lectotype and, most importantly, it is exactly how *Onchidium* slugs (in the strict sense) look like when they are preserved without relaxation or when they are alive but disturbed.

##### Type material

**(*Onchidium nigrum*).** Holotype, by monotypy (ZMB 22749). One specimen 40/30 mm, completely dissected (by Plate) and empty. There is a vial with destroyed pieces of the digestive system (mostly the digestive gland and the intestine).

##### Type material

**(*Elophilus ajuthiae*).** Three syntypes (MNHN 22965) 20/17, 20/15 and 20/14 mm. All three specimens, dissected by Labbé, are now empty. Only a few destroyed pieces of the digestive system remain in a vial.

##### Additional material examined.

**Malaysia**, Peninsular Malaysia, Kuala Sepatang, 04°50.434N, 100°38.176E, 18 July 2011, 1 specimen (42/24 [DNA 971] mm), leg. B. Dayrat & T. Goulding, [station 27, old forest with tall, old *Rhizophora* trees, high in the tidal zone (ferns), following boardwalk in educational preserve, reached a creek lower in the tidal zone, with mud] (USMMC 00006); **Brunei Darussalam**, Mentiri, Jalan Batu Marang, 04°59.131N, 115°01.820E, 29 July 2011, 3 specimens (33/18 [#1], 20/16, and 16/13 [DNA 1048] mm), leg. T. Goulding & S. Calloway, [station 36, old mangrove with tall *Rhizophora* trees with high roots and *Thalassina* mounds] (BDMNH); **Philippines**, Bohol, Inabanga, 10°04.255'N, 124°04.416'E, 13 July 2014, 3 specimens (from 30/20 [DNA 3251] to 25/17 mm), leg. J. Comendador, B. Dayrat & T. Goulding, [station 187, mostly *Nypa* palms with *Thalassina* mounds] (PNM 041199); Inabanga, 10°04.432'N, 124°04.691'E, 13 July 2014, 1 specimen (27/17 mm), leg. J. Comendador, B. Dayrat & T. Goulding, [station 188, old forest, untouched for about 30 years, mostly *Avicennia*, many old logs] (PNM 041200); Mabini, 09°51.532'N, 124°31.685'E, 17 July 2014, 1 specimen (35/25 mm), leg. J. Comendador, B. Dayrat & T. Goulding, [station 194, narrow forest on the edge of fish ponds, tall *Rhizophora* and *Avicennia* trees, many old logs, muds of different types] (PNM 041201); Mabini, 09°51.402'N, 124°30.982'E, 18 July 2014, 4 specimens (from 35/28 [#1] and 35/22 [#2, DNA 3363] to 12/9 mm), leg. J. Comendador, B. Dayrat & T. Goulding, [station 195, narrow forest with tall trees on the edge of fish ponds, cemented ditches between the mangrove patches and the ponds] (PNM 041202); **Vietnam**, Can Gio, 10°24.171'N, 106°53.960'E, 10 July 2015, 1 specimen (30/20 [DNA 5602] mm), leg. T. & J. Goulding, [station 221, hard mud by a small road and then a steep bank to the soft, deep mud, *Avicennia* and some *Rhizophora* trees spread out] (ITBZC IM 00001); Can Gio, 10°26.703'N, 106°53.694'E, 12 July 2015, 7 specimens (from 55/35 to 25/16 mm; 44/30 [DNA 5605], 35/25 [#1] mm), leg. T. & J. Goulding, [station 223, margin of mangrove by creek, shrimp ponds behind the mangrove, fairly high intertidal] (ITBZC IM 00002); Can Gio, 10°27.620'N, 106°53.316'E, 17 July 2015, 3 specimens (from 28/18 to 12/8 mm), leg. T. & J. Goulding, [station 231, open mangrove with large *Avicennia* trees, soft mud, some old logs] (ITBZC IM 00003); Can Gio, 10°24.157'N, 106°53.950'E, 19 July 2015, 1 specimen (20/12 mm), leg. T. & J. Goulding, [station 233, hard mud by a small road and then a steep bank to the soft, deep mud, open forest of *Avicennia* and *Rhizophora*, rocks and gravel on side of mangrove] (ITBZC IM 00004); **China**, Macau, 3 specimens (42/28 to 32/28 mm), leg. Heynemann, (SMF 333591/3).

##### Distribution

(Fig. 2). Malaysia, Sabah (type locality of *Vaginulus stuxbergi*); Brunei Darussalam (present study, new record); Malaysia, Peninsular Malaysia (present study, new record); Philippines, Bohol (present study, new record); Vietnam (present study, new record); Thailand (type locality of *Elophilus ajuthiae*); China (present study; Sun et al. 2014, one individual misidentified as “*Onchidium struma*” *nomen nudum*). A specimen of *Onchidium stuxbergi* was found in Singapore (in the mangrove by the Mandai River) but was unfortunately lost. The presence of *Onchidium stuxbergi* was also documented (as *Onchidium nigram*, which is a spelling mistake) in a guide to the mangroves of Singapore (Ng and Sivasothi 2002: 115). The type locality of *Onchidium nigrum* simply was cited as “Borneo,” which could be anywhere on the island in Indonesia (Kalimantan) or Malaysia (Sabah or Sarawak). Our record in Macau is the northernmost (22°10'N) confirmed locality on the coast of southern China.

##### Habitat

(Fig. 9). The habitat of *Onchidium stuxbergi* is very similar to that of *Onchidium typhae*: directly on mud (not soft, i.e. mud that is not very watery), on muddy trunks, old logs, lobster mounds, and even under *Nypa* leaves (Philippines). However, *Onchidium stuxbergi* was not observed on mud as soft as the mud on which *Onchidium typhae* was found in West Bengal.

##### Abundance.

*Onchidium stuxbergi* is a rare species. Only one individual was found in Malaysia (where 18 mangrove sites were explored), three individuals at one site in Brunei Darussalam (7 sites), nine individuals at four sites in Bohol (17 sites), and 12 individuals at four sites in Vietnam (19 sites). Even though it will need to be confirmed in the future, it seems that *Onchidium stuxbergi* tends to be slightly more common (although still rare, overall) in more northern latitudes (Vietnam and Philippines).

##### Color and morphology of live animals

(Fig. 10). Live animals are not always covered with mud and the color of their dorsum can normally be seen. The dorsum is brown, with no particular pattern. Exceptionally, it can be almost black. The hyponotum varies between grayish and yellowish, and sometimes even greenish. It occasionally bears conspicuous black dots. The foot is bright orange, which is different from the two other species described here. In a tiny specimen (12 mm in length, St. 195/17), the foot was pale yellow. The long ocular tentacles are cream to brown distally, and darker proximally. The head is brown to black, with many evenly distributed white markings. The morphology of live specimens is similar to that of *Onchidium typhae*. The only difference is that the central papilla with a few dorsal eyes is prominent in *Onchidium stuxbergi*. Crawling individuals normally measure between 30 and 40 mm in length. Preserved specimens no longer display the distinct color traits seen in live animals. The color of preserved animals is meaningless and uninformative. The background color of the notum is brown. Some individuals, including old ones (SMF 333491) bear a few irregular, darker markings. The hyponotum and the foot of preserved animals are homogenously white.

##### Internal anatomy

(Figs 11–13). Examples of radular formulae are: 70 × 75-1-75 in USMMC 00006 (42 mm long), 50 × 68-1-68 in BDMNH #1 (33 mm long), and 70 × 80-1-80 in PNM 041202 #1 (35 mm long). The intestine is long, narrow, and of the so-called “type III,” characterized by the fact that a transverse line can intersect the intestine eight times.

The oviduct is narrow, short, and straight. The hollow spine of the penial accessory gland is slender and slightly curved. It measures between 0.5 (USMMC 00006) and 1.4 mm (PNM 041202 #2) in length, and between 20 (USMMC 00006) and 35 (PNM 041202 #2) µm in diameter. The diameter of the opening at its tip is nearly 10 µm. The hollow spine does not open directly into the vestibule. Instead, the end of the tube of the accessory gland is a disc which is more or less flat (approximately 0.3 mm in diameter) and does not seem to bear distinct conical papillae. The hollow spine must thus go through that disc in order to be outside and shared with the partner.

The penial sheath is long (to the posterior third of the visceral cavity) and coiled in a few spirals. In less mature individuals, the coils may not be as marked. The retractor muscle is short and inserts into the posterior third of the visceral cavity. There is an additional retractor muscle attaching the anterior portion of the penial sheath to the left wall of the visceral cavity, near the buccal mass. In some individuals, that left additional retractor muscle is very thick and strong. The deferent duct is highly convoluted with many loops, but less so in immature specimens. The penis is elongated, round, narrow, and hollow. Its diameter is less than 200 µm. Its distal part is covered with hooks. When the penis is retracted inside the penial sheath, the hooks are inside the tube-like penis. During copulation, the penis is exerted like a glove and the hooks are then on the outside. Hooks are very densely packed inside the penis, with multiple, irregular rows of an average of 15 hooks around the circumference of the penis. Hooks are conical, slender, sharply pointed, and measure from 40 µm up to 300 µm in length. The longer they are the more slender they are.

##### Distinctive diagnostic features.

Externally, *Onchidium stuxbergi* differs from other *Onchidium* species by the color of the foot, which is bright orange (see below the dichotomous identification key, before the final conclusion). Internally, *Onchidium stuxbergi* is the only *Onchidium* species known so far (and the only onchidiid species, for that matter) with a strong, additional retractor muscle attaching the anterior penial sheath to the left, anterior wall of the visceral mass, near the buccal mass.

##### Remarks.

The status of *Onchidium stuxbergi* has been problematic from the start because Westerlund unknowingly based his original description on specimens that are part of two distinct species (see Type materials, above): eleven former syntypes (now paralectotypes) are *Platevindex* and another former syntype (now the lectotype) is an *Onchidium* (in the strict sense, as defined here). Two years after the original description, Westerlund (1885) again published the description of *Vaginulus stuxbergi*, as a new species again. Although that contribution is not the original description, its figure 2 helps confirm that *Vaginulus stuxbergi* is an onchidiid and, most importantly, illustrates the one former syntype (here designated as the lectotype) that is part of *Onchidium*. Therefore, even though the brief and vague description may be confusing (because it is based on two different species), the illustration makes the identification absolutely clear, hence our decision to designate the illustrated specimen as the lectotype. Note that Westerlund did not describe any internal characters.

However, as a direct consequence of Westerlund’s ambiguous original description and type material, many authors have proposed synonymies between *Onchidium stuxbergi* and some species names that clearly belong to *Platevindex*. Those cases are briefly discussed here, but they will be discussed in more detail in our revision of the genus *Platevindex*. Labbé (1934: 235) and Hoffmann (1928: 88) both regarded *Onchidium stuxbergi* as a *Platevindex* (as *Oncis stuxbergi*) and suggested that *Onchidella condoriana* Rochebrune, 1882 and *Oncis inspectabilis* Plate, 1893, could be synonyms of *Onchidium stuxbergi*. *Onchidium condoriana* and *Oncis inspectabilis* clearly belong to *Platevindex* (types were examined) and are not synonyms of *Onchidium stuxbergi*. Hoffmann (1928: 88) also regarded *Onchidium coriaceum* Semper, 1885, as a synonym of *Onchidium stuxbergi* but it actually is a valid name of the genus *Platevindex*. Finally, Hoffmann (1928: 88) suggested that *Onchidium ponsonbyi* Collinge, 1901, could possibly be a synonym of *Onchidium stuxbergi* but it is very unlikely because *Onchidium ponsonbyi* is a terrestrial species known from 850 to 1,050 meters high at Mt Penrissen, Borneo. *Onchidium ponsonbyi* likely belongs to the genus *Semperoncis* Starobogatov, 1976.

There is no doubt that *Onchidium nigrum*, which is only known from the holotype, belongs to the genus *Onchidium* as re-defined here: the mantle of the preserved holotype bears the typical papillae of *Onchidium*. Also, Plate described both a rectal gland and an accessory gland, which are found in all three known *Onchidium* species. Plate did not mention the presence of an additional, left, retractor muscle for the penial sheath. He only mentioned that the insertion of the retractor muscle is of “type II” (i.e., near the pericardium). According to Plate, the penial hooks are from 14 to 87 µm long and the spine of the penial accessory gland is 1.2 mm long. The penial hooks observed here are from 40 to nearly 300 µm in length. It is possible that Plate, who observed only one specimen, could not fully evaluate the variation of penial hooks. Also, penial hooks are extremely challenging to extract and observe without SEM. However, Plate’s description of the penial accessory gland spine is fully compatible with our observations (from 0.5 to 1.2 mm long). Finally, Plate described the intestine loops of *Onchidium nigrum* as of a unique and exceptional pattern, which he referred to as “type III.” The latter, as illustrated by Plate (1893: plate 8, fig. 31a) is slightly more coiled than what was observed for the present study, but they are basically identical patterns. That intestinal pattern is absent from *Onchidium typhae* but it has also been observed in some species from other genera. Given that Plate did not know the color of the live animal, it will never be known whether it matched the diagnostic color of the foot that was observed for our specimens (bright orange). According to our data, *Onchidium stuxbergi* is distributed from Malaysia to Vietnam and the Philippines and therefore mostly encompasses Borneo. As a result, the synonymy of *Onchidium nigrum* and *Onchidium stuxbergi* is warranted, even though it cannot be completely excluded that *Onchidium nigrum* could refer to an *Onchidium* species remotely endemic to the south east of Borneo.

The three syntypes described as *Elophilus ajuthiae* by Labbé (1935) were earlier identified by him as *Onchidium nigrum* (Labbé, 1934). His first identification was supported by a pattern of intestinal loops (Plate’s “type III”) only known from *Onchidium nigrum*. Labbé changed his mind after the observation of what he thought were tiny dorsal gills (“microbranchies”) in those three syntypes from Thailand. Indeed, according to Labbé’s (1934) onchidiid classification, dorsal gills are only found in the Dendrobranchiatae (which includes genera such as *Peronia*, *Paraperonia*, and *Scaphis*) while all other onchidiids (such as *Onchidium*, *Onchidella*, *Platevindex* and *Onchidina*), the Abranchiatae, lack dorsal gills. The three specimens from Thailand (with gills) could not belong to *Onchidium* (no gills) and, as a result, Labbé created a new species name and a new genus name for those specimens with an intestine of “type III” and dorsal gills. Labbé’s new genus *Elophilus*, preoccupied, was replaced by Starobogatov (1976) by *Labella*. Those three syntypes from Thailand were re-examined for the present study; unfortunately, they are mostly empty. A few destroyed pieces of the intestine system remain but they are completely useless. However, the mantle clearly does not bear any “microbranchies” (i.e., microgills). It is very likely that Labbé’s first intuition was correct and that he was just looking at large *Onchidium* papillae retracted within the mantle. Those three specimens from Thailand are part of *Onchidium nigrum*, which means *Onchidium stuxbergi*. Unfortunately, Labbé did not describe the male copulatory complex in detail and so the sizes and shapes of the penial accessory spine and of the penial hooks are unknown. However, our specimens from Vietnam suggest that there is only one species of *Onchidium* distributed in the region of the South China Sea, *Onchidium stuxbergi*. Naturally, it cannot be excluded that the Gulf of Thailand actually hosts a distinct species; however, there is nothing in Labbé’s description supporting that hypothesis. As a result, the synonymy of *Labella ajuthiae* with *Onchidium stuxbergi* is warranted. Also, Labbé was confused about the type locality of *Onchidium nigrum* because he claimed that “Plate’s unique specimen came from Borneo (Guam)” (Labbé 1934: 223, our translation) and that “Plate’s unique specimen came from the Marianna Islands” (Labbé 1935: 312, our translation). Borneo is with no doubt the type locality of *Onchidium nigrum*. Finally, Labbé’s claim that *Labella ajuthiae* lived in fresh water was unfounded. Even though the specimens were collected far inland, it was still in brackish water and under the influence of the tides (see above, Type localities).

The name *Onchidium struma*, introduced by Qiu (1991) and used occasionally in the Chinese literature to refer to some onchidiids from the coast of China (e.g., Shen et al. 2006; Sun et al. 2014), is a *nomen nudum* (to our knowledge, *Onchidium struma* has not been formally described as a new species). A survey of the diversity of onchidiids from China based on molecular data was recently published (Sun et al. 2014). The sequences of the specimens identified as *Onchidium* “*struma*” by Sun et al. were all included in our analyses here, and that name appears in two distinct species units (Fig. 1), which demonstrates that the name *Onchidium* “*struma*” used by Sun et al. referred to two distinct species. One of their species, identified here as *Onchidium reevesii*, is mostly subtropical and is distributed from 22°30’ to 34°36’ latitude north along the coast of China. So far, it seems to be endemic to China. In the data set of Sun et al., *Onchidium reevesii* is represented by eight individuals (under the name *Onchidium* “*struma*”). Note that in that same contribution, Sun et al. (2014) apply the name *Paraoncidium reevesii* to a different species but that is also a misidentification. The species they refer to as *Paraoncidium reevesii* obviously cannot be *Onchidium reevesii*, but it does not belong to *Paraoncidium* either. The other species referred to as *Onchidium* “*struma*”, identified here as *Onchidium stuxbergi*, is tropical and, in China, is only found in the southernmost coastline. In the data set of Sun et al., only one individual from Hainan Island (19°56'N) can be safely referred to as *Onchidium stuxbergi*. Another individual in their data set (from Hong Kong, at 22°28'N) is problematic because its CO1 and 16S sequences give contradictory results, and so it is likely that one of those sequences is a mistake. The three specimens examined for the present study from Macau (SMF 333591/3) are the northernmost confirmed locality of *Onchidium stuxbergi* in China at 22°10'N.

#### Onchidium reevesii

Taxon classificationAnimaliaSystellommatophoraOnchidiidae

(J.E. Gray, 1850)

Fig. 14

Onchidella reevesii J.E. Gray, 1850: 117, pl. 181, fig. 5–5a; Hoffmann 1928: 103.
Onchidium reevesii : Semper 1885: 290.

##### Type locality.

China. *Onchidella reevesii* was not described by Gray. That name, which appears on page 117, simply referred to figures 5 and 5a of the plate 181). On page 117, there is no indication of the geographic origin, and there is no indication of geographic origin on the label of the holotype either. However, on page 36, those same figures are referred to as “*Onchidium* —. Mr. Reeve’s drawings. China,” clearly indicating that the animal illustrated on the fig. 5 (and 5a for the ventral side) of the plate 181 is from China. Hoffmann (1928) also accepted China as the type locality.

##### Type material.

**Figure 14.**
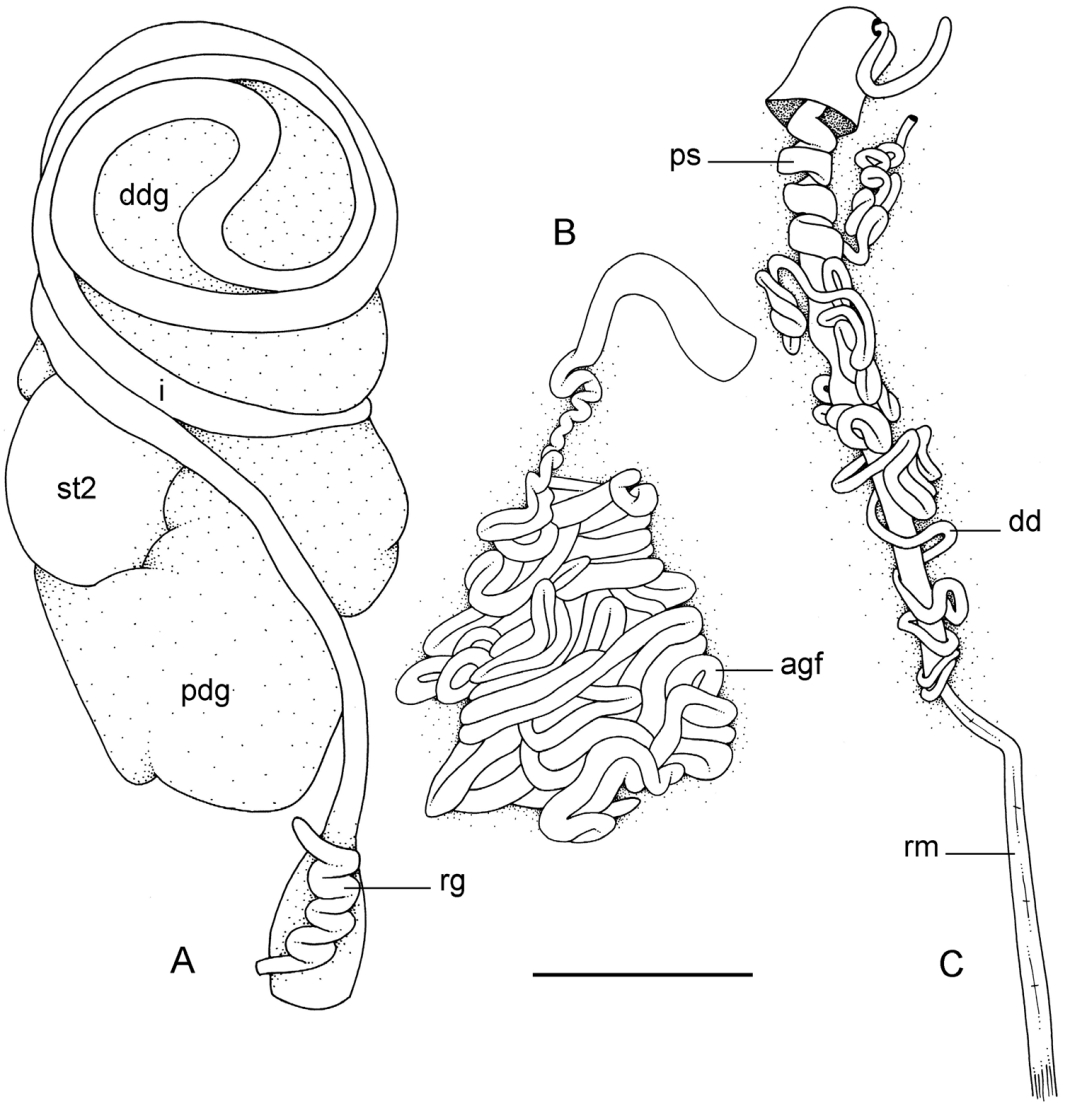
Internal anatomy, holotype, *Onchidium reevesii* (NHMUK 20160036). **A** Digestive system, dorsal view, scale bar 7.5 mm **B** Reproductive system, anterior, penial accessory gland, scale bar 3 mm **C** Reproductive system, anterior, penial sheath, scale bar 5 mm. Abbreviations: agf, accessory gland flagellum; dd, deferent duct; ddg, dorsal lobe of digestive gland; i, intestine; pdg, posterior lobe of the digestive gland; ps, penial sheath; rg, rectal gland; rm, retractor muscle; st2, stomach chamber 2.

Holotype (43/25 mm), by monotypy (NHMUK 20160036). The label says “? Holotype *Onchidella reevesii* Gray 1850,” but there is no reason to think it is not the holotype because the specimen fits perfectly the original illustration and the label also does refer to “Gray, Figs. Moll. Anim., IV, 1850, p. 117” where the name *Onchidella reevesii* was first published. The holotype is in excellent condition. It was opened dorsally prior to the present study so several key features could easily be checked in the digestive system as well as the male anterior parts, which are all drawn here (Fig. 14). The posterior reproductive parts (far less critical for species identification) were not removed from the visceral cavity (because it would have required our destroying the posterior region of the holotype) and so they are not illustrated here.

##### Distribution

(Fig. 2). China (type locality). Based on sequences misidentified as *Onchidium* “*struma*” (a *nomen nudum*) by Sun et al. (2014) and re-analyzed here, *Onchidium reevesii* is found on most of the coastline of mainland China (except for southernmost and northernmost latitudes), from 22°30’ to 34°36’ of latitude north.

##### Internal anatomy

(Fig. 14). The intestine of the holotype is between a type II and a type III, because a transverse line can intersect the intestine six times (four times for the type II as in *Onchidium typhae*, and eight times for the type III as in *Onchidium stuxbergi*). Although observed, the hollow spine was not extracted. At the end of the flagellum of the accessory gland, there is a flat disc which distally protects the hollow spine. The penial sheath is coiled in spirals. The retractor muscle is shorter than the penial sheath and inserts at the posterior end of the visceral cavity. There is no additional left retractor muscle. The deferent duct is highly convoluted with many loops. The penis of the holotype was not extracted for the present study.

##### Distinctive diagnostic features.

Externally, *Onchidium reevesii* differs from other *Onchidium* species by its ventral color, i.e., a whitish hyponotum and foot (see the dichotomous key below). Internally, the spirally coiled penial sheath of *Onchidium reevesii* is not diagnostic (as in *Onchidium stuxbergi* and *Onchidium typhae*). However, *Onchidium reevesii* lacks the additional, anterior, left retractor muscle of the penial sheath that is unique to *Onchidium stuxbergi* (the only species with which *Onchidium reevesii* may be shown later to overlap in the southernmost part of its distribution).

##### Remarks.

Semper transferred *Onchidella reevesii* to *Onchidium* probably by default (i.e., as a non-*Onchidella* species) because he did not give any explanation and he did not examine any new material. At any rate, it just so happens that *Onchidium reevesii* is the correct combination, because the notum of the type specimen bears the long papillae that are typically found in *Onchidium* as defined here. The presence of a rectal gland and of an accessory penial gland also supports that *Onchidella reevesii* is an *Onchidium*, even though those traits are found in other genera and even though the accessory penial gland can be both present and absent within a genus. That being said, a rectal gland and an accessory gland are found in all other *Onchidium* species. There is a small disc at the distal end of the accessory gland of the holotype of *Onchidium reevesii*, a structure which we found so far only in *Onchidium*.

Hoffmann (1928: 69) placed *Onchidium reevesii* in the genus *Oncis* (i.e., *Platevindex*) with a question mark but, in the same publication (Hoffmann 1928: 103), accepted it as an *Onchidella* also. Hoffmann did not have access to new material and he does not seem to have examined the type because he did not comment on it. Britton (1984) then used the new combination *Paraoncidium reevesii* based on material that was sent to him from Hong Kong but without examining the type material of *Onchidium reevesii*. However, Britton’s identification was erroneous because he described *Paraoncidium reevesii* as lacking both a rectal gland and an accessory penial gland, while both glands are actually present in the holotype of *Onchidium reevesii*. Also, *Paraoncidium* Labbé, 1934 actually is a junior synonym of *Onchidina* Semper, 1885, and thus refers to a different clade.

Sun et al. (2014) adopted Britton’s (1984) work and their use of the name *Paraoncidium reevesii* is a misidentification because it refers to a species with no rectal gland and no accessory penial gland, based on our own dissections of that species. The molecular study of Sun et al. (2014) shows that there are eight onchidiid species in China. Unfortunately, their identifications are erroneous (at the specific and/or generic levels). However, by including their sequences in our comprehensive regional data set and due to our own dissections it is possible to know what those species are as well as their internal anatomy.

There are actually only two species in China with both a rectal gland and an accessory penial gland, and both species belong to the genus *Onchidium*. In the study by Sun et al. (2014), those two species are confused under a single name, *Onchidium* “*struma*”, which is a *nomen nudum*. One of those two species, *Onchidium stuxbergi*, is restricted to the extreme southernmost latitudes of the coast of China (up to 22°10'N) but is also distributed in Malaysia (Sabah, Peninsula), Vietnam, Brunei, and the Philippines (see above *Onchidium stuxbergi*). The other species, *Onchidium reevesii*, seems to be endemic to China and is distributed along nearly the entire coast of China (from 22°30’ to 34°36’), except for the southernmost and northernmost latitudes. It makes sense that *Onchidium reevesii* applies to the species that is the most widely distributed in China because its type locality (“China”) had a much higher chance to fall within the range of *Onchidium reevesii* compared to that of *Onchidium stuxbergi*.

*Onchidium reevesii* and *Onchidium stuxbergi*, of which the distribution ranges do not seem to overlap, differ externally and internally. A few pictures of live animals of *Onchidium* “*struma*” from Shangai, northern China (Shen et al. 2006: fig. 1), show that the foot of *Onchidium reevesii* is whitish (the foot of *Onchidium stuxbergi* is bright orange). Internally, the holotype of *Onchidium reevesii* is lacking the additional, left, retractor muscle that is exclusively diagnostic of *Onchidium stuxbergi*. The spiral coils of the penial sheath of the holotype of *Onchidium reevesii* are compatible with the illustration (although fuzzy) of the anterior male apparatus of specimens misidentified as *Onchidium* “*struma*” from Shangai (Wu et al. 2007: fig. 1). However, spiral coils of the penial sheath are not diagnostic of *Onchidium reevesii* because they are also found in *Onchidium typhae*. Finally, there are fewer loops in the intestine of the holotype of *Onchidium reevesii* than in *Onchidium stuxbergi* (of type III) and, based on our data, the intestine of a species cannot be of different types.

### Identification key

A key is provided here to help identify the three known species of *Onchidium*. The key is based on external characters because they are the most readily available. However, internal diagnostic features also distinguish the species (see species descriptions). DNA sequences provided in the present contribution can also help clear any potential confusion, because, to date, DNA sequences of *Onchidium* have yielded unambiguous results.

**Table:** 

1	The foot is bright orange	***Onchidium stuxbergi*** (known distribution: Peninsular Malaysia, Thailand (Gulf of Thailand), Vietnam, eastern Borneo, Philippines, southernmost tropical China).
–	The foot is not bright orange	**2**
2	The hyponotum is white and the foot is whitish	***Onchidium reevesii*** (known distribution: subtropical China, from 22°30’ to 34°36’)
–	The hyponotum (and the foot) is not white but varies between grayish and yellowish, and sometimes even greenish	***Onchidium typhae*** (known distribution: Bengal, Andaman Islands, Malaysia, Singapore).

## Discussion

Naturally, new species of *Onchidium* may be discovered in the future. However, our data currently support the existence of only three species. It is worth pointing out that *Onchidium* is not found in eastern Indonesia and is also absent in recent collections from Madang, Papua New Guinea, by the Paris Museum (MNHN). *Onchidium* were not found either on the western coast of India, Madagascar, or Mauritius. Its geographic distribution, as suggested by the present results, thus ranges from north-eastern India to the Philippines, including the Strait of Malacca, Singapore, Thailand, Vietnam, eastern Borneo, and China. So, even though *Onchidium* may be found from additional localities in the future (e.g., *Onchidium stuxbergi* should occur in Palawan, Philippines), the distribution of *Onchidium* proposed here may be close to its actual distribution.

Based on current data, the distribution ranges of *Onchidium stuxbergi* and *Onchidium reevesii* do not overlap. If they do overlap, it may simply be over a very small area around Hong Kong. The geographic distribution of *Onchidium reevesii* is typically subtropical (from 22°30’ to 34°36'N) and tolerance for different water temperatures may have largely participated in the speciation between *Onchidium stuxbergi* and *Onchidium reevesii*, which are most closely related in our phylogenetic analysis (Fig. 1). That the individuals of *Onchidium stuxbergi* in the Philippines (Bohol) show some genetic divergence from the rest of the species can be easily explained due to their relative isolation from the rest of the species, centered about the South China Sea.

Several authors in the past (e.g., Plate 1893; Hoffmann 1928; Labbé 1934; Britton 1984) have commented on the variation of some of the anatomical traits that seem important for the higher classification of the Onchidiidae (e.g., rectal gland, penial accessory gland, position of the male aperture, and pattern of the intestinal loops). However, all those comments have been confusing because both genus and species identifications have remained highly problematic. Even though it still is too early to draw some general conclusions regarding the variation of anatomical characters across all onchidiids, it is appropriate to comment on their variation within the genus *Onchidium*. The rectal gland and the penial accessory gland (and its hollow spine) are present in all three species. This does not mean that the presence/absence of these two structures do not vary within other genera, it simply means that these structures are always present in *Onchidium*. The male aperture is always inferior to the right eye tentacle, slightly to its left (i.e., in dorsal view, which means that the male aperture actually is in between the two eye tentacles). The patterns for the loops of the intestine (the types I, II, III, etc., as defined by Plate and Labbé) need to be commented upon. Labbé created a distinct genus name (*Labella*, here a synonym of *Onchidium*) almost exclusively based on the presence of an intestine of type III in *Labella ajuthiae* (here a synonym of *Onchidium stuxbergi*). However, our results show that both the type II and the type III are found in *Onchidium* (type II in *Onchidium typhae*, type III in *Onchidium stuxbergi*). The intestine of the holotype of *Onchidium reevesii* is intermediary between a type II and a type III. However, the intestine type does not seem to vary within each species. It will certainly be very interesting to look at the distribution of these characters across all onchidiids, and map them onto a general phylogeny.

Because the new limits to the genus *Onchidium* are much more restricted than its traditional meaning, many specific names traditionally combined with *Onchidium* must be combined with different generic names (Dayrat 2009). Those names will be dealt with the systematic revision of each clade of Onchidiidae in our future contributions. Here, however, comments are being provided on existing *Onchidium* species names that are regarded as *nomina dubia*, i.e., names which have been validly published but that should simply be ignored because their application is doubtful.

*Onchidium aberrans* Semper, 1885 is a *nomen dubium* because its type locality (Singapore) was mentioned as uncertain in the original description. Also, no type material could be located. *Onchidium griseo-fuscum* (Tapparone-Canefri, 1874), originally described as *Onchidella griseo-fusca* from Singapore and for which no type material could be located, could not be associated with any of the species we collected in Singapore. It could belong to *Peronia* but it is uncertain. As a result, it is here regarded as a *nomen dubium*. *Onchidium hardwickii* (J. E. Gray, 1850) was originally described as *Onchidella hardwickii* from an unknown locality and is thus regarded here as a *nomen dubium*. The holotype (by monotypy, MNHN) of *Onchidium harmandianum* Rochebrune, 1882, originally described from the Côn Đảo archipelago, off southern Vietnam, is a piece of tissue that may not even be part of an onchidiid slug. Because its original description is too brief and uninformative, it is regarded as a *nomen dubium*. We explored this archipelago and no species could be associated with that name. *Onchidium planatum* Quoy and Gaimard, 1824, originally described from Guam, is a *nomen dubium*: the lack of type material (not located), a vague original description, and the lack of illustration make it nearly impossible for it to be re-identified. More importantly, it is not even sure, based on the original description, that it actually was an onchidiid (after all, *Onchidium secatum*, from the same publication and by the same authors, is clearly not an onchidiid). *Onchidium tabularis* (Tapparone-Canefri, 1883), originally described as an *Onchidella tabularis* from Wokam, Aru Islands, Indonesia, is a *nomen dubium* due to the lack of type material (not located), the lack of illustration, and a useless written description. Tapparone-Canefri suggested that *Onchidium tabularis* might refer to the same species as *Onchidium planatum*, but the latter is also a *nomen dubium*. Additionally, none of the species that we collected from Kei Islands (which is very close to Aru Islands) could be linked to *Onchidium tabularis*. *Onchidium tricolor* Simroth, 1918, also described from Aru Islands, is a *nomen dubium* because the type material could not be located, there is no internal description, and the drawings of the external morphology are not informative enough. Finally, *Onchidium trapezoideum* Semper, 1885 is a *nomen dubium* because the type locality is unknown.

The present contribution illustrates well some of the complicated and relatively common issues faced in taxonomy and the possible ways to address them within an integrative approach. Integrative taxonomy is more than simply comparing morphological data and molecular data: nomenclatural issues are at the core of integrative taxonomy (Dayrat 2005). Nomenclatural issues are not something one can think about after species units have been delineated. Nomenclatural issues need to be considered from the very beginning of a taxonomic study. For instance, as our field work was being planned, type localities were included as our top priorities and were actually visited as often as possible. That allowed us to include specimens from type localities in our molecular and morphological data sets, and easily find available names for the species units being delineated. Specimens from type localities are not indispensable to obtain well-delineated species units, but they are critical to name them. For instance, in the present study, it would have been very challenging to determine the status of the names *Onchidium typhae* and *Onchidium stuxbergi* without newly-collected specimens from West Bengal and Brunei Darussalam, their respective type localities. The good news is that taxonomic work becomes possible and much easier if type materials are examined, original descriptions are carefully studied, and new specimens are collected from (as many) type localities (as possible). The not-so-good news is that there is no such thing as a quick taxonomic study because addressing all these issues can take a great deal of time and expertise.

## Supplementary Material

XML Treatment for Onchidium

XML Treatment for Onchidium typhae

XML Treatment for Onchidium stuxbergi

XML Treatment for Onchidium reevesii

## References

[B1] Baker HB (1938) Nomenclature of Onchidiidae. The Nautilus 51: 85–88. doi: 10.5962/bhl.part.10914

[B2] Britton KM (1984) The Onchidiacea (Gastropoda, Pulmonata) of Hong Kong with a worldwide review of the genera. Journal of Molluscan Studies 50: 179–191.

[B3] Buchannan F (1800) An account of the *Onchidium*, a new genus of the class of vermes, found in Bengal. Transactions of the Linnean Society of London 5: 132–134. [pl. 5]

[B4] Collinge WE (1901) On the anatomy of a collection of slugs from N.W. Borneo. Transactions of the Royal Society of Edinburgh 40: 295–312. [pls 1–3]

[B5] Cuvier G (1804) Mémoire sur l’Oncidie, genre de mollusques nuds, voisin des limaces, et sur une espèce nouvelle, *Onchidium peronii*. Annales du Muséum national d’histoire naturelle 5: 37–51. [1 pl]

[B6] Dayrat B (2005) Towards Integrative Taxonomy. Biological Journal of the Linnean Society 85: 407–415. doi: 10.1111/j.1095-8312.2005.00503.x

[B7] Dayrat B (2009) Review of the current knowledge of the systematics of Onchidiidae (Mollusca: Gastropoda: Pulmonata) with a checklist of nominal species. Zootaxa 2068: 1–26.

[B8] Dayrat B Conrad M Balayan S White TR Albrecht C Golding R Gomes S Harasewych MG Frias Martins AM de (2011a) Phylogenetic relationships and evolution of pulmonate gastropods (Mollusca): new insights from increased taxon sampling. Molecular Phylogenetic and Evolution 59: 425–437. doi: 10.1016/j.ympev.2011.02.014 10.1016/j.ympev.2011.02.014 21352933

[B9] Dayrat B Zimmermann S Raposa M (2011b) Systematic revision of the Onchidiidae from the Tropical Eastern Pacific. Journal of Natural History 45: 939–1003. doi: 10.1080/00222933.2010.545486

[B10] Dey A (2006) Handbook on Mangrove Associate Molluscs of Sundarbans. Zoological Survey of India, Kolkata, 96 pp.

[B11] Guindon S Gascuel O (2003) A simple, fast, and accurate algorithm to estimate large phylogenies by maximum likelihood. Systematic Biology 52: 696–704. doi: 10.1080/10635150390235520 14530136 10.1080/10635150390235520

[B12] Gray JE (1850) Figures of molluscous animals selected from various authors. Etched for the use of students by M.E. Gray. Longman, Brown, Green and Longmans, London, vol. 4, 219 pp.

[B13] Hoffmann H (1928) Zur Kenntnis der Oncidiiden. Zoologische Jahrbücher (Jena) 55: 29–118.

[B14] Klussmann-Kolb A Dinapoli A Kuhn K Streit B Albrecht C (2008) From sea to land and beyond. New insights into the evolution of euthyneuran Gastropoda (Mollusca). BMC Evolutionary Genomics 8: 57. doi: 10.1186/1471-2148-8-57 10.1186/1471-2148-8-57 PMC2287175 18294406

[B15] Labbé A (1934) Les Silicodermés (Labbé) du Muséum d’Histoire Naturelle de Paris. Première partie: Classification, nouvelles ou peu connues. Annales de l’Institut Océanographique de Monaco 14: 173–246.

[B16] Labbé A (1935) Sur une forme nouvelle de Silicoderme, *Elophilus ajuthiae* nov. gen. nov. sp. Bulletin de la Société Zoologique de France 60: 312–317.

[B17] Milne I Wright F Rowe G Marshal DF Husmeier D McGuire G (2004) TOPALi: Software for Automatic Identification of Recombinant Sequences within DNA Multiple Alignments. Bioinformatics 20: 1806–1807. doi: 10.1093/bioinformatics/bth155 14988107 10.1093/bioinformatics/bth155

[B18] Ng PKL Sivathosi N (2002) A guide to the mangroves of Singapore II. Singapore Science Center, Singapore, 168 pp.

[B19] Plate L von (1893) Studien über opisthopneumone Lungenschnecken, II, Die Oncidiidien. Zoologische Jahrbücher, Anatomie und Ontogenie der Thiere 7: 93–234. [pl. 7–12]

[B20] Qiu LY (1991) Morphology and habit of *Onchidium struma* along the coast of Jiangsu and Shanghai in China. Chinese Journal of Zoology 26: 33–36.

[B21] Quoy JRC Gaimard JP (1824) Zoologie. Voyage autour du Monde entrepris par ordre du Roi, sous le ministère et conformément aux instructions de S. Exc. M. Le Vicomte du Bouchage, secrétaire d’État au Département de la Marine, exécuté sur les corvettes de S. M. l’Uranie et la Physicienne, pendant les années 1817, 1818, 1819 et 1820; publié par M. Louis de Freycinet. Pillet Aîné, Paris, 672 pp. [vol. 1], 96 pls [vol. 2].

[B22] Rafinesque CS (1815) Analyse de la nature ou tableau de l’univers et des corps organisés. [self-published by the author], Palerme, 223 pp. doi: 10.5962/bhl.title.106607

[B23] Rochebrune AT (1882) Documents sur la faune malacologique de la Cochinchine et du Cambodge. Bulletin de la Société philomatique, Paris 6: 35–74.

[B24] Ronquist F Huelsenbeck JP (2003) MrBayes 3: Bayesian phylogenetic inference under mixed models. Bioinformatics 19: 1572–1574. doi: 10.1093/bioinformatics/btg180 12912839 10.1093/bioinformatics/btg180

[B25] Semper C (1885) Dritte Familie, Onchidiidae. In: Semper C (Ed.) Reisen im Archipel der Philippinen, Wissenschaftliche Resultate, III (7). C.W. Kreidel, Wiesbaden, 251–290. [pl. XIX–XXVII]

[B26] Shen H Chen H Chen X Dai X Shi Z Ran F (2006) Experimental study on the reproductive biology of *Onchidium struma*. Journal of Fisheries of China 30: 753–760.

[B27] Simroth H (1918) Über Einige Nacktschnecken von Malayischen Archipel. Abhandlungen der Senckenbergischen Naturforschenden Gesellschaft, Frankfurt am Main 35: 259–306. [pl. XX]

[B28] Singkran N (2015) Water quality and TMDL determinations for the Chaopraya River, Thailand. In: Chan D (Ed.) Environmental Science and Information Application Technology. CRC Press, Boca Raton, 27–32. doi: 10.1201/b18559-6

[B29] Starobogatov YI (1976) Composition and taxonomic position of marine pulmonate mollusks. Soviet Journal of Marine Biology 4: 206–212.

[B30] Stoliczka F (1869) The malacology of Lower Bengal. Journal of the Asiatic Society of Bengal 38: 86–111. [pls 14–15]

[B31] Sun B Chen C Shen H Zhang K Zhou N Qian J (2014) Species diversity of Onchidiidae (Eupulmonata: Heterobranchia) on the mainland of China based on molecular data. Molluscan Research 34(1): 62–70. doi: 10.1080/13235818.2013.868860

[B32] Tamura K Strecher G Peterson D Filipski A Kumar S (2013) MEGA6: Molecular Evolutionary Genetics Analysis version 6.0. Molecular Biology and Evolution 30(12): 2725–2729. doi: 10.1093/molbev/mst197 24132122 10.1093/molbev/mst197 PMC3840312

[B33] Tapparone-Canefri C (1874) Zoologia del viaggio intorno al globo della regia fregata Magenta duranto gli anni 1865–68. Malacologia (Gastropodi, Acefali e Brachiopodi). Paravia, Torino, 152 pp.

[B34] Tapparone-Canefri C (1883) Fauna malacologica delle Nuova Guinea e delle Isole adiacenti. Annali del Museo Civico di Storia Naturale, Genova 19: 1–313.

[B35] Westerlund CA (1885) Land- och sötvatten-mollusker, insamlade under Vega-Expeditionen. Vega-Expeditionens Veteskapliga Iakttagelser 4: 191–192. [pl. 4]

[B36] Wu Y Shen H Dai X Fu J Yang Y (2007) Studies on histology of reproductive system of *Onchidium* sp. Journal of Fishery Sciences of China 14(7): 17–21.

